# A survey of models composed of graph neural networks and large language models for molecular science

**DOI:** 10.1093/bioinformatics/btag387

**Published:** 2026-06-15

**Authors:** Natàlia Segura-Alabart, Francesc Serratosa

**Affiliations:** Departament d’Enginyeria Informàtica i Matemàtiques, Universitat Rovira i Virgili, Tarragona, Catalonia 43007, Spain; Departament d’Enginyeria Informàtica i Matemàtiques, Universitat Rovira i Virgili, Tarragona, Catalonia 43007, Spain

## Abstract

**Motivation:**

Graphs have been demonstrated to have an impressive ability to keep the structural and semantic properties of chemical compounds and are therefore widely used in molecular modeling. Moreover, Graph Neural Networks (GNNs) and Large Language Models (LLMs) have achieved outstanding results in predicting molecular properties and generating textual descriptions given graphs or texts, respectively. Recently, some mathematical models have been presented for molecular science applications, which combine the GNNs ability to capture the structural and semantic information and the generative ability of LLMs. However, these models are dispersed across the literature and vary in architecture, input–output design, and application scope, making it difficult to systematically compare them or to identify suitable approaches for specific research objectives.

**Results:**

This paper classifies recent GNN–LLM models and summarizes them with the aim of providing guidance for research involving their use or the development of new models. We present tables that depict the properties and parameters of the models, as well as their associated chemical computational applications. In addition, a new model classification is presented to help researchers define the models they use or future ones. Finally, we quantitatively compare these models based on their reported experimental results.

## Graphical Abstract

**Graphical Abstract:**
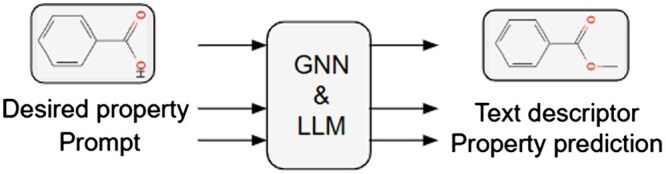

## 1 Introduction

Graphs have been used for decades to represent and analyze complex relationships in real-world applications, from social networks to chemical compounds and biological data (Garcia-Hernandez *et al.* 2020). Currently, Graph Neural Networks (GNNs) (Zhou *et al.* 2020, Zhang *et al.* 2021, Waikhom and Patgiri 2023) have demonstrated remarkable achievements in various applications involving the classification or regression of objects represented as attributed graphs. Examples include Graph Convolutional Networks (GCN) (Kipf and Welling 2016), Graph Transformers (GT) (Yun *et al.* 2022), Graphormers (Ying *et al.* 2021), Graph Isomorphism Network (GIN) (Xu *et al.* 2019) and Graph AutoEncoders (GAE) (Serratosa 2025). In all these models, there exists an implicit assumption of a relationship between the node attributes and the local structure (Wang *et al.* 2022).

Recently, Large Language Models (LLMs) have achieved tremendous success in various domains (Zhao *et al.* 2024). In some cases, they have also been used in graph-related tasks and have surpassed traditional GNN (Waikhom and Patgiri 2023). While their primary focus has been on text sequences, there is a growing interest in enhancing the multi-modal capabilities of LLMs to enable them to handle diverse data types, including graphs. For this reason, some models have appeared that integrate LLM and GNN, with the aim of extracting the most from the ability of GNNs to deal with structural knowledge and the ability of LLM to generate new coherent knowledge.

The fusion of graphs and LLMs has proven effective in various graph-related tasks (Jin *et al.* 2024, Li *et al.* 2024). Integrating LLMs with traditional GNNs offers mutual benefits, thereby enhancing graph learning capabilities. By incorporating LLMs, GNNs gain richer node features that encapsulate both structural and contextual elements. Conversely, LLMs are adept at encoding textual information but often struggle with the structural relationships inherent in graph data. Combining GNN with LLM leverages the strengths of both to create a more robust and comprehensive approach to graph learning.

In the context of molecular science applications, several models based on the integration of GNN and LLM have recently emerged (Edwards *et al.* 2021, Su *et al.* 2022, Abdine *et al.* 2023, Liu *et al.* 2023a, 2023b, Shi *et al.* 2023, Zhao *et al.* 2023, Liu *et al.* 2024, Cao *et al.* 2025, Liu *et al.* 2025). These models have been applied to several downstream tasks such as molecular property prediction, classification, and molecule generation. In particular, these tasks often rely on human-understandable language inputs rather than explicit formulated numerical targets or handcrafted features. For instance, a model can generate a new chemical compound based on a known compound in response to prompts such as “generate a new compound similar to this one but with higher solubility” or “generate a new compound similar to this one but bigger.” Although certain aspects of these tasks could, in principle, be performed by GNN alone with carefully engineered inputs, such natural-language prompts are only possible through the integration of the latest LLMs with GNN architectures.

The aim of this paper is to analyze and summarize recent models that integrate GNN and LLM applied to molecular science. The authors did not want to list all LLM and GNN models, but to list which combinations have been presented and to list which architectures and codes are ready to be used. The authors are aware of the existence of LLM surveys applied to graphs (Jin *et al.* 2024, Li *et al.* 2024), as well as the existence of competitive molecular models that do not use GNNs (Christofidellis *et al.* 2023, Liu *et al.* 2023c, Sun *et al.* 2025). The aim of this paper is not to publish a new general survey, but to provide a domain-focused resource for molecular sciences and to be a tool for rapidly learning which LLM-GNN model fits the best in a specific problem. The models applied to molecular science from Jin *et al.* (2024) have been selected, which is a seminal paper. This survey reviews GNN-LLM models that incorporate existing publicly available LLMs rather than designing them from scratch as part of the overall architecture. Interestingly, other models have also emerged for protein-drug binding affinity prediction that integrate GNNs and LLMs but rely on custom-built LLMs instead of publicly available ones. The most common approach in these cases is the use of transformer-based architectures (Xie *et al.* 2025, Cucco 2026, Li *et al.* 2026). Such models are not included in this article, as the main objective of this work is to analyze the advantages and limitations of integrating different established LLMs into GNN-based frameworks. Finally, all the experimental results have been extracted from the papers that explain these models.

The structure of the paper reflects this objective. Section *Methods* is divided into four subsections. Subsection *Classification of methods based on GNN-LLM integration for molecular analysis* presents two taxonomies proposed by other authors for classifying models that integrate GNN and LLM, and then proposes a new taxonomy specifically tailored for molecular science applications. Subsection *Model overview* provides a comprehensive overview of the analyzed models. Subsection *Molecular Tasks in GNN–LLM models* summarizes the various molecular tasks that these models are capable of performing, together with detailed experimental results compiled from the literature. Subsection *Specific definition of models with GNN-LLM integration for molecule analysis* outlines the specific architectures for each of the models identified in the literature, providing a general overview of their design. Section *Conclusions* concludes the paper. The Supplementary Material includes both general and detailed graphical schemes of the models. It also provides a summary of the experiments conducted in the selected papers and discusses current and future models.

## 2 Methods

In this survey, we use the term LLM in a broad methodological sense, referring to models trained to learn patterns over discrete sequences using supervised objectives. These sequences may correspond to natural language text or to domain–specific symbolic representations such as molecular strings (e.g. SMILES). However, it is important to distinguish between general–purpose LLMs (General-LLM), which are designed for conversational text generation, and representation–specific LLM, which are trained on structured biological or chemical sequences (Molecular-LLM). Examples of the latter include protein language models, genome language models, and chemical language models operating on SMILES or SELFIES.

### 2.1 Classification of methods based on GNN-LLM integration for molecular analysis

For a reader interested in the current LLM architectures, Zhao *et al.* (2024) presents an in-depth analysis and new taxonomy. However, the analysis of LLM *per se* is not within the scope of this paper. Interestingly, two different taxonomies of GNN-LLM integration have been proposed (Jin *et al.* 2024, Li *et al.* 2024).

In Li *et al.* (2024), a survey of models that integrate GNN and LLM for any type of application is presented. Roughly speaking, the following taxonomy is presented, in which the application is not considered but the architecture of the model:

1. **LLM as an enhancer**: These models use LLMs to generate explanations, which are new attributes in the graph nodes. Then, the models predict some properties based on GNNs that use these graphs. Therefore, the first step is an LLM and the second step is a GNN.
2. **LLM as a predictor**: Contrary to the previous class, the prediction is carried out by the LLM instead of a GNN. Here, the initial structural information is converted into a text thanks to a GNN. Therefore, the first step is a GNN followed by the LLM in the second step.
3. **GNN–LLM alignment**: There are no first and second steps. Both models, GNN and LLM work in parallel with different types of data (structural or text). Through a contrastive learning process, they generate similar embeddings when the introduced information needs to be aligned or paired.

In Jin *et al.* (2024), there is also a taxonomy, whose main focus is the type of graphs introduced into the models. Thus, in the first taxonomy level, they classified the models by the type of graph. And in the second level, by the three categories previously depicted. The taxonomy directly relates the type of graph to the problem to be solved.

1. **Pure graphs**: Pure graphs are graphs that do not have attributes on their nodes or edges. They are a universal representation used to address a wide range of algorithmic problems such as shortest paths and flow networks, which are strongly connected to real-world applications.
2. **Text-attributed graphs**: Text-attributed graphs are graphs with textual information at node-level. These types of graph exist ubiquitously in the real world. Some examples range from social networks to chemical compounds. Learning the model based on such graphs requires the model to encode both the textual information associated with the nodes and edges and the structure information lying inside the input graph.
3. **Text-paired graphs**: Text-paired graphs are graphs with graph-level textual information, meaning that the textual data is associated with the entire graph, rather than with individual nodes or edges. This category is similar to the category **GNN–LLM alignment** presented in the previous taxonomy. These methods contain an LLM component for text encoding and a GNN component for structure encoding. These two components are served equally and are trained iteratively or in parallel. LLMs and GNNs can mutually enhance each other, since LLMs can provide textual signals to GNNs, while GNNs can deliver structure information to LLMs.

**Figure 1:**
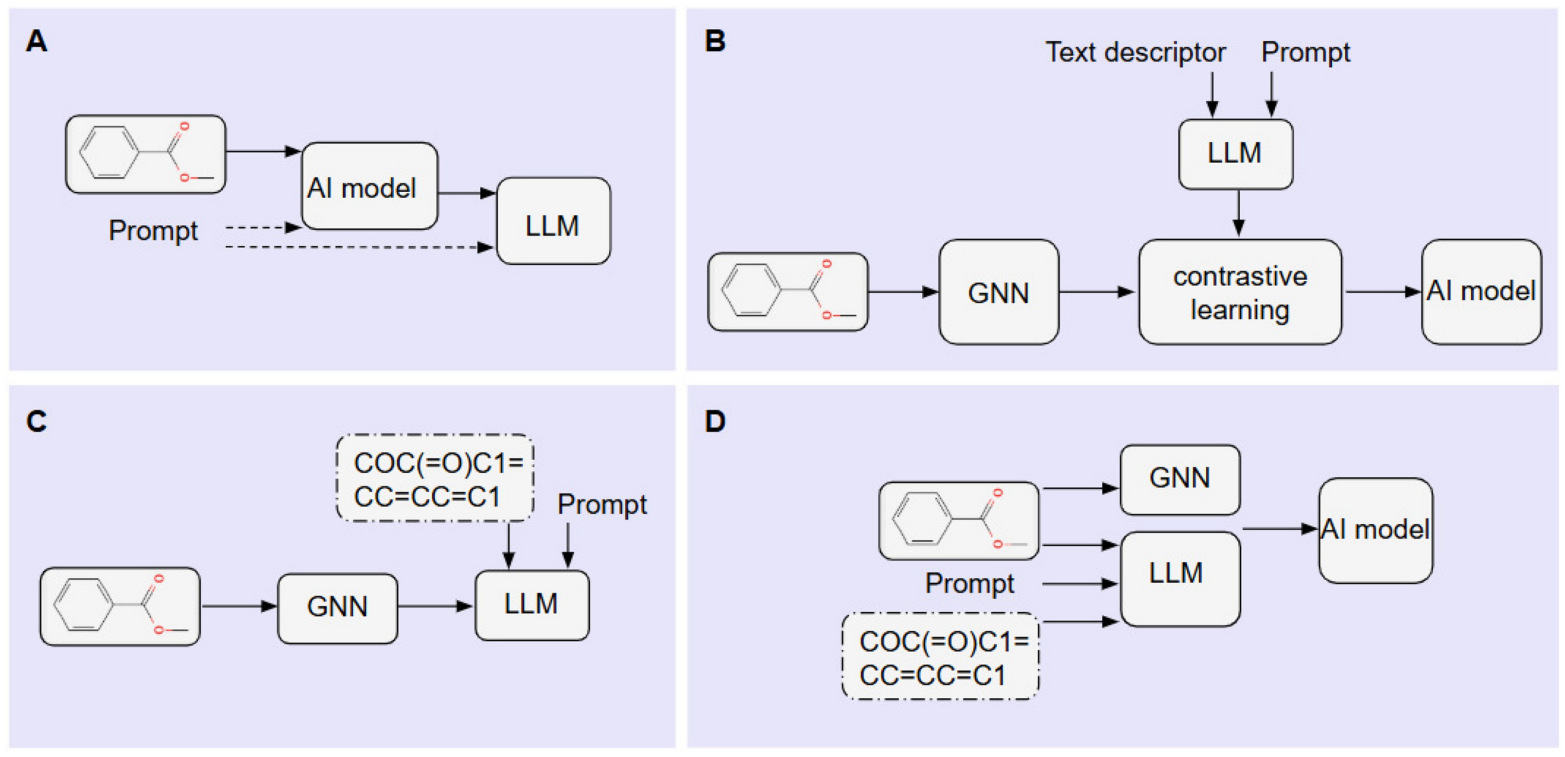
GNN-LLM classification (A) GNN-LLM sequence, (B) GNN-LLM align, (C) GNN-LLM serie include, and (D) GNN-LLM parallel include.

Due to the fact that this article is dedicated to models applied to molecular analysis and property prediction we have developed a specific taxonomy for this purpose. In this context, we detail the architecture used in chemical analysis applications, without considering the type of graphs as all of them are attributed. Our taxonomy consists of three classes, which are schematically represented in Fig. 1.

1. **GNN-LLM sequence**: These models involve two steps: The first step transforms the molecule, which is represented as a graph, into a sequence of nodes or tokens, and it is done by an AI model that can be a GNN, a Neural Network (NN) or a Graph-Text Neural Network (GTNN). The second step parses this sequence into an LLM, which generates the predicted output. The AI Model is usually trained, but this is not the case for the LLM, which can either be frozen or trained. Examples are GIMLET (Zhao *et al.* 2023), ReLM (Shi *et al.* 2023), Prot2Text (Abdine *et al.* 2023), and Llamole (Liu *et al.* 2025).
2. **GNN-LLM include**: These models leverage the advantages of GNNs to incorporate inherent structural characteristics and dependencies present in graph data directly into the LLMs, allowing the LLMs to be structure-aware. Integrating GNN representations into LLMs requires training both the GNN and the LLM. Various strategies have been proposed to fuse the structural patterns learned by GNNs with the contextual information captured by LLMs. These strategies encompass the LLM to be used after the GNN (Serie Include) or at the same time (Parallel Include). Examples are InstructMol (Cao *et al.* 2025) and MolCA (Liu *et al.* 2023b) for Serie Include and GIT-Mol (Liu *et al.* 2024) for Parallel Include.
3. **GNN-LLM align**: These models aim to directly inject molecular structural information into the LLM. To this end, explicit structural descriptors are provided as primary inputs. The fusion of direct structural inputs and GNN–derived representations can be realized either sequentially, where a GNN is first applied to the molecular graph and its output is subsequently incorporated into the LLM alongside explicit structural descriptors, or in parallel, where direct structural inputs and GNN–based features are processed simultaneously and jointly fused within the LLM framework (Fig. 1C and D, respectively). Representative examples of this class include Text2Mol (Edwards *et al.* 2021), MoMu (Su *et al.* 2022), and MoleculeSTM (Liu *et al.* 2023a).

In the rest of the paper, a detailed explanation of the models provided as examples in each class is presented.

### 2.2 Model overview

This survey presents a review of 10 models that integrate GNN and LLM applied to drug analysis and discovery found in the current literature. Table 1 provides a summary of the key characteristics of each model, including the name of the model, year of publication, the specific GNN and LLM architectures used, their classification approach described in Section *Classification of methods based on GNN-LLM integration for molecular analysis*, and references to the original articles. The term “Tuning” (column seven in Table 1) indicates whether the parameters of the LLM require adjustment. The snowflake symbol indicates that the LLM is frozen, meaning fine-tuning is not permitted, while the fire symbol signifies that the LLM allows fine-tuning. “Prompt” (column eight in Table 1) indicates the use of text-formatted prompts within the LLMs, either manually or automatically generated. It is distinguished between general–purpose LLMs (G-LLM) and representation–specific molecular LLMs (M-LLM). Although all are based on Transformer architectures, their training data and intended semantics differ substantially. In addition, Supplementary Table 18 includes links to the corresponding code for each model.

**Table 1:** Overview of GNN-LLM models.

Model	Year	GNN	G-LLM	M-LLM	Class	Tuning	Prompt	Reference
GIMLET	2023	GT	T5		Sequence	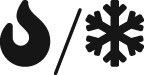	Yes	Zhao *et al.* (2023)
ReLM	2023	GCN/TAGCN	Vicuna/ChatGPT		Sequence	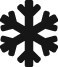	Yes	Shi *et al.* (2023)
Prot2Text	2024	RGCN	ChatGPT		Sequence	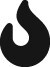	Yes	Abdine *et al.* (2023)
Llamole	2025	GIN & GraphDiT	LLaMA/Mistral/Qween/Granite/T5		Sequence	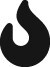	Yes	Liu *et al.* (2025)
InstructMol	2024	GIN	Vicuna		Serie Include	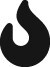	Yes	Cao *et al.* (2025)
GIT-Mol	2024	GIN	SciBERT	MolT5	Parallel Include	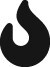	Yes	Liu *et al.* (2024)
MolCA	2023	GINE	Galactica	MolT5	Serie Include	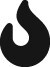	Yes	Liu *et al.* (2023b)
Text2Mol	2021	GCN	SciBERT		Align	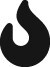	No	Edwards *et al.* (2021)
MoMu	2022	GIN	BERT		Align	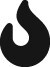	No	Su *et al.* (2022)
MoleculeSTM	2024	GIN	BERT	MegaMolBART	Align	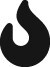	Yes	Liu *et al.* (2023a)

G-LLM: General LLM. M-LLM: Molecular LLM. The “/” in the GNN and LLM columns indicates that the model can use any of the listed options, while “&” indicates components used jointly. For MoleculeSTM, the LLM options can either be used simultaneously or individually. 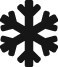: Frozen, 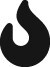: LLM fine-tuning.

Section *GNN and LLM Explanations* of the Supplementary Material provides explanations of the used GNNs (third column of Table 1)—GCN, TAGCN, RGCN, GT, GIN, GINE, and GraphDiT—and the used LLMs (fourth and fifth columns of Table 1)—T5, MolT5, Vicuna, ChatGPT, BERT, SciBERT, Galactica, MegaMolBART, LLaMA, Mistral, Qwen, and Granite.

### 2.3 Molecular tasks in GNN–LLM models

Table 2 shows the downstream molecular science tasks for these models in the first column. A number is used to indicate whether a specific chemical task is performed by a model, instead of the traditional “*X*.” This number is the row in Supplementary Table 3 that relates this specific combination in Section *Comparative Evaluation Framework: Databases, Metrics, Baselines, and Published Results.* Ten different tasks are depicted in the experimental section of related papers. Moreover, we have mapped these tasks to the most closely related Machine Learning (ML) function. The link between the chemical perspective and the machine learning perspective is crucial to understanding the models we want to describe. We realized that an article may describe only one model, but it is utilized for several tasks. Logically, a downstream task may be addressed in several papers. The remainder of this section summarizes each of these tasks.

**Table 2:** Summary of 10 downstream tasks addressed by the analysed papers and their relation to the machine learning functions.

Chemical task	ML function	GIMLET	ReLM	Prot2Text	Llamole	InstructMol	GIT-Mol	MolCA	Text2Mol	MoMu	MoleculeSTM
Molecule-to-Descriptor retrieval	Graph-to-text retrieval							14		19	24
Descriptor-to-Molecule retrieval	Text-to-graph retrieval							15	18	20	25
Molecular property prediction	Graph regression	1				6					
Molecular property classification	Graph classification	2				7	10			21	26
Chemical reaction prediction	Graph transformation prediction		3		5	8					
Generative molecule description	Generative graph description			4		9	11	16		22	
Generative molecule naming	Generative graph naming							17			
Text-to-molecule generation	Text-to-graph generation						12			23	
Molecule editing	Graph-to-graph generation										27
Molecule image naming	Graph image-to-text						13				

Numbers are the rows in Supplementary Table 3 that relate the specific combinations.

#### 2.3.1 Molecule to descriptor retrieval

This task retrieves a descriptor, for instance, a text description (natural language) or an SMILES representation, from a given dataset containing pairs of molecules and their corresponding description (text or SMILES). The objective is to identify the most relevant descriptor that accurately matches the given molecule.

#### 2.3.2 Descriptor to molecule retrieval

This task retrieves a molecule from a database given a descriptor such as a SMILES or a human-intelligible sentence such as “The molecule is a simple sugar called glucose, which consists of six carbon atoms, twelve hydrogen atoms, and six oxygen atoms arranged in a ring structure with multiple hydroxyl (-OH) groups.”

#### 2.3.3 Molecular property prediction

Given a molecule represented in either SMILES format or utilizing both their structural and sequential information, and a text query such as “Given the structure of molecule X, what is its predicted solubility in water (logS value)?,” the model predicts the addressed property.

#### 2.3.4 Molecular property classification

Molecular property classification is analogous to property prediction. Nevertheless, the key distinction lies in the model’s output, which provides a categorical classification, such as a binary “yes” or “no” or the class to which it belongs, rather than a continuous value.

#### 2.3.5 Chemical reaction prediction

This task involves three different prediction approaches, which can be conceptualized in the general format of a chemical reaction: reactant → reagent → product. These approaches allow for exploration at different points within the reaction pathway:

1. Forward Reaction Prediction: Given the input reaction and its associated conditions, the model generates a potential product of the reaction.
2. Reagent Prediction: Given the reactants and products, the goal is to identify the appropriate catalysts, solvents, or auxiliary substances required for the specific chemical reaction (reagents).
3. Retrosynthesis: This approach is the inverse of forward reaction prediction. In this case, the model is provided with the product, and it predicts the possible reactants that could have led to the formation of that product.

It is important to note that in this task only one of the possible solutions is provided. For example, although multiple reagents may be viable for the given reaction, only one is predicted.

ReLM, Llamole and InstructMol are capable of performing this task. However, ReLM is limited to forward reaction prediction, and its output is a SMILES representation of the predicted product. In contrast, Llamole only performs rethrothynthesis. InstructMol, on the other hand, is capable of performing all three tasks.

In machine learning, this task is defined as graph transformation prediction, as it involves starting with graphs, and the model predicts a new graph based on the input graphs.

#### 2.3.6 Generative molecule description

Given a molecule and a text query such as “Can you please give me a brief description of this compound?,” the model will provide a text explanation that addresses the specific query. A simplified answer could be “The molecule is a natural product found in or produced by the bacterium *Streptomyces griseus*.” Additionally, for the Prot2Text model, the model can also accept the text sequence as an input.

#### 2.3.7 Generative molecule naming

Given a molecule and a text query such as “Can you please provide the name of this compound?,” the model generates a name for that compound in formats such as SMILES, IUPAC or any other standard molecular naming convention.

This task is close to the description of the previously described generative molecule; however, we have classified it as a different task because providing a description of a compound is not equivalent to actually naming it. The former involves generating a textual explanation in the natural language of the compound, whereas the latter requires the generation of a formal name according to established conventions.

#### 2.3.8 Text-to-molecule generation

This task generates novel molecules that are not present in existing molecular datasets but adhere as closely as possible to a provided description. For example, a basic textual prompt such as “The molecule is a type of simple sugar that serves as a primary source of energy for many living organisms” may lead the model to generate a molecular representation like “C(C1C(C(C(C(O1)O)O)O)O)O,” which corresponds to the SMILES notation of the glucose molecule. In this scenario. the model is assumed not to have been explicitly trained on, or previously aware of, the glucose molecule. Rather, the model generates the structure based solely on the description provided.

#### 2.3.9 Molecule editing

The aim of this task is to modify the chemical structure of molecules, focusing on transformations such as functional group change and scaffold hopping (Liu *et al.* 2023a). The model generates new molecular structures based on a given molecule.

#### 2.3.10 Molecule image naming

This task is similar to the Text-to-molecule generation task, although accepts a 2D molecular image as input instead of a text. The model generates molecular representations in SMILES format. Although image transformation is not typically addressed by GNN, we have included this task because one of the selected papers considers it.

### 2.4 Specific definition of models with GNN-LLM integration for molecule analysis

This part presents general definitions of the models introduced in Section *Model overview* to facilitate a clearer understanding of their main tasks and overall structure. To this end, we develop both a general and a model-specific schema for each approach, outlining the tasks they perform, using a consistent format across all models. This standardized representation is intended to simplify and clarify the comparisons between them.

A comprehensive evaluation of the baseline models is provided in the Supplementary Material due to space reasons. Experimental results compiled from prior studies are newly organized according to our categories of chemical prediction and generation tasks. For completeness, the Supplementary Material also includes a consolidated recompilation of tasks (represented as numbers 1–27 in Table 2) and the datasets used to evaluate each model, the associated evaluation metrics, and the baseline methods considered for comparison (Supplementary Table 3). A summarized assessment of the strengths and limitations of the 10 analyzed models is also presented in Supplementary Table 17.

#### 2.4.1 GIMLET

The Graph Instruction-based Molecule Zero-shot Learning (GIMLET) adopts a distance-based position embedding that simultaneously utilizes both molecular structures and the instructions provided by the prompt as input. GIMLET is designed to predict or classify specific molecular properties (Supplementary Fig. 2A). In the process of positional encoding for the graph, GIMLET defines the relative position of two nodes as the shortest distance between them within the graph, which has been widely used in the literature of GTs (Ying *et al.* 2021).

In contrast to other presented models, GIMLET can simultaneously learn both the molecular structure representation and instructions from the prompt, without requiring additional encoding GNNs. This approach is referred to as the Graph-Text Neural Network (GTNN). A more detailed overview of GIMLET is presented in Supplementary Fig. 9.

#### 2.4.2 ReLM

The REaction Prediction Language Model (ReLM) starts with a list of reactants, represented as molecular 2D graphs, along with the associated reaction conditions. ReLM is designed to predict the results of chemical reaction products (Supplementary Fig. 2B).

To achieve this, as shown in Supplementary Fig. 10, the model first generates several high-probability product candidates using a GNN. These candidates, along with relevant examples in-context, and the reaction conditions, are then used as a prompt in a multiple-choice question format to an LLM. The LLM utilizes this data to facilitate accurate predictions of chemical reaction products. In-context examples are extracted from a reactant-product dataset, which is queried using top-N nearest neighbours to identify reactant-product pairs such that the reactants are similar to the input reactants.

#### 2.4.3 Prot2Text

Prot2Text begins with the amino-acid sequence, provided as raw text. Prot2Text is a multimodal generative framework designed to produce free-text functional descriptions of proteins, moving beyond traditional classification schemes that rely solely on predefined labels or ontologies (Supplementary Fig. 3).

As illustrated in Supplementary Fig. 11, Prot2Text adopts an encoder–decoder architecture that leverages the complementary strengths of GNNs, NNs and transformer-based LLMs. The protein structural information is computed by AlphaFold2 developed by DeepMind (Google). First, a GNN encodes the protein graph to capture structural and relational patterns among residues. In parallel, the amino-acid sequence is processed by a transformer encoder, specifically the ESM-2 model bio (Lin *et al.* 2022), which is a protein language model trained on sequence data. Interestingly, ESM-2 shows that structural information emerges directly from sequence–only training. These two modalities are projected into a shared latent space and fused to form a unified representation that summarizes both local and global protein properties. This fused representation is then provided as conditioning context to an LLM decoder, which generates coherent, natural-language descriptions of the protein’s biological function.

#### 2.4.4 Llamole

The Large LAnguage model for MOLEcular discovery (Llamole) is a multimodal LLM that unifies text and graph generation for inverse molecular and polymer design with retrosynthetic planning (Supplementary Fig. 2D). The model combines a foundational LLM (such as LLaMA, Mistral, Qwen, Gravite, T5) with GNN, including GIN and Graph DiT. SMILES strings are converted into 2D molecular graphs, which are then encoded by the GNN and processed by the LLM in conjunction with the input prompt. Through controlled activation of the LLM, the model can generate molecular structures, polymers, and textual explanations in a single sequence.

Furthermore, Llamole capabilities extends beyond single-step retrosynthesis by enabling multi-step retrosynthetic tasks, thereby supporting more complex and iterative design processes. This multi-step retrosynthetic capability represents a key characteristic of the model.

#### 2.4.5 InstructMol

InstructMol effectively enables the LLM to interpret molecular representations and generalize across a range of molecular tasks. As a multi-modal instruction model, it utilizes the same architecture for molecular property prediction and classification, and generative molecule description (Supplementary Fig. 4A). The model accepts a 2D representation of the molecule, along with its sequential information encoded as an adapted SMILES notation, known as SELFIES (Krenn *et al.* 2020), and a prompt. The 2D molecular structure is introduced into a GNN to generate a graph embedding, which is then passed into a neural network (NN) for transformation into graph tokens. Along with the other inputs, the prompt, and the sequential information of the molecule (if provided), these graph tokens are directly fed into the LLM. The LLM then combines the graph-structured data with the textual information to predict molecular properties, perform classification, or generate molecular descriptions. A more detailed overview of InstructMol for these three tasks is provided in Supplementary Fig. 12.

InstructMol can predict chemical reactions from any of the three different perspectives: forward reaction prediction, reagent prediction, and retrosynthesis (Supplementary Fig. 4B–D). The prediction of chemical reaction also incorporates sequential information.

#### 2.4.6 GIT-Mol

GIT-Mol is a multi-modal LLM that integrates 2D molecular graph, image, and text information for molecular science (Supplementary Fig. 5). At its core, GIT-Mol employs GIT-Former, an innovative architecture adapted from BLIP-2’s Q-Former (Li *et al.* 2023) that efficiently aligns and fuses all types of data it processes into a unified latent space. This architecture enables adaptation to a wide range of scenarios, allowing for substitution of images or graphs with other modalities. Once the various types of molecular data are fused, they are passed through a NN to perform specific tasks. Depending on the type of molecular data input, GIT-Mol executes the corresponding task. For example, when provided with a molecular graph and a SMILES representation, GIT-Mol classifies a specific molecular property. A detailed representation of the types of input data and their corresponding tasks is provided in Supplementary Fig. 13.

Both text-to-molecule generation and molecule image naming do not rely on a GNN. These tasks are included to demonstrate the full range of capabilities of GIT-Mol. In particular, GIT-Mol is the only model presented in this paper capable of processing image data.

#### 2.4.7 MolCA

Molecular Graph-Language Modeling with a Cross-Modal Projector and Uni-Modal Adapter (MolCA) shares a similar architecture with GIT-Mol, utilizing a Q-Former as the cross-modal projector. The Q-Former map the output from the GNN encoder to the input text of the LLM, thereby enabling the extraction of text-relevant molecule features. As a result, MolCA is capable of performing both molecule-to-descriptor and descriptor-to-molecule retrieval tasks (Supplementary Fig. 6A and B). Furthermore, by incorporating a Uni-Modal Adapter into the LLM, MolCA can generate molecule descriptions and accurately produce corresponding IUPAC names based on 2D molecular graphs and SMILES representations (Supplementary Figs. 6C and D and 14).

#### 2.4.8 Text2Mol

Text2Mol introduces a cross-modal attention-based model to fuse graph and textual embeddings early on, using a transformer decoder where the LLM output is the source sequence and the GNN output is the target sequence. This setup allows the attention mechanism to learn multi-modal associations. The model treats molecules as a language with its own unique grammar, enabling it to combine text and molecular data. Text2Mol retrieves the molecule corresponding to a given text description from a database, without requiring any reference textual information, (Supplementary Fig. 2C). Molecules can be represented as SMILES strings, molecular graphs, or equivalent representations. A more specific schema of Text2Mol is shown in Supplementary Fig. 15.

#### 2.4.9 MoMu

Molecular Multimodal (MoMu) model employs the GNN called GIN with several layers and a final average pooling of all node features to encode the molecular 2D graphs. The atom number and atom chirality are node features of the graph. There is an edge in the graph if the two atoms share a bond. Moreover, an LLM encoder for text data and/or SMILES representations is also applied. It is a Bert model, which is widely used as a feature extractor in natural language processing. These encoders are jointly trained using contrastive learning on a dataset of paired molecular graphs and their corresponding textual descriptions, aligning their representations in a shared feature space. The model’s design facilitates the integration of structural and textual data. A general schema of MoMu is shown in Supplementary Fig. 7. Due to the generalization ability of the GNN and LLM encoders, MoMu can be adapted to various downstream tasks such as cross-modality retrieval, molecule caption, property prediction, and text-to-graph molecule generation, and thus effectively facilitates molecular-related scientific exploration.

#### 2.4.10 MoleculeSTM

MoleculeSTM adopts paired graph embeddings and text embeddings to perform contrastive learning. These components work together to align molecular structures with their corresponding textual or sequence-based representations within a shared feature space. Its architecture integrates a GNN encoder to process molecular 2D graphs and two separate LLM encoders—one specifically for text data and the other for SMILES representations. Consequently, MoleculeSTM is capable of performing both molecule-to-descriptor and descriptor-to-molecule retrieval tasks (Supplementary Fig. 8A and B). The model can accept molecular 2D graphs and SMILES representations as input; however, when only SMILES are provided, the GNN is not utilized. Similarly, in the molecular property classification task, only one of the encoders—the GNN or the LLM- is used, depending on the input type, as shown in Supplementary Figs. 8C and 16.

Additionally, MoleculeSTM is capable of molecule editing (Supplementary Fig. 8D). The edition is composed of two main steps. In the first, called space alignment, the model aligns the representation space of a pretrained molecule generation model and the representation space of MoleculeSTM. In the second, called latent optimization, the model learns a latent representation that might be similar to both input molecules and textual descriptions.

## 3 Conclusions

The aim of this survey is to present a tool for developers or researchers to know which are the current models that they can use or which ones can be the basis for future models. We have presented a survey of models that integrate GNN and LLM applied to chemical compound property prediction, compound generation, or textual description generation, among others. We have classified these models and explained their properties. Moreover, we have detailed the GNNs and LLMs they use and also the databases that have been used or created to train them.

From Section *Comparative Evaluation Framework: Databases, Metrics, Baselines, and Published Results*, it is evident that hybrid models that combine GNNs and LLMs currently achieve the most promising results within the evaluated benchmarks. This success is largely due to their ability to combine the strengths of both approaches: GNNs are effective at learning from molecular structures, while LLMs provide strong semantic and contextual reasoning. In particular, the prompting abilities of LLM have been shown to enhance model performance, suggesting that prompt design remains an important aspect of high model performance.

One notable trend among top-performing models is their use of 3D molecular representations, either alone or combined with 2D information, rather than traditionally 2D formats such as those extracted from SMILES, suggesting its potential importance, although systematic comparisons remain limited. This shift reflects the growing recognition of spatial molecular information in the ability to accurately capture chemical properties.

Another important observation is that the choice of GNN architecture must be carefully tailored to the specific task. Effective models are those that can represent not only atoms and bonds but also more complex molecular substructures and relationships. However, training these models can be computationally expensive and, in some cases, unrealistically time-consuming. This challenge has led to an increased interest in approaches that use frozen LLMs, which can significantly accelerate training and reduce computational cost.

Despite ongoing efforts by the authors of these models to create specialized datasets, there remains a lack of standardized, curated databases tailored to specific tasks. This limitation presents a notable barrier to reproducibility, model comparison, and widespread adoption.

Looking ahead, future research is likely to focus on the development of more efficient, task-specific hybrid models, potentially incorporating methods such as Density Functional Theory (DFT) to improve predictive accuracy. Within the context of GNN–LLM hybrid models reviewed in this survey, there is also likely to be an emphasis on pretraining large models on extensive molecular datasets before fine-tuning them for specific tasks since from the evaluated models only one does not allow pretraining (ReLM). In addition, integration of multimodal data, including molecular graphs, chemical text, spectral data, and images, will likely become increasingly important in enhancing model robustness and versatility.

Addressing the computational cost of model training will remain a priority, with efforts directed toward improving scalability and enabling the integration of these models into high-throughput experimental workflows. Leveraging unlabeled molecular data through contrastive learning techniques may further enhance model generalization, especially when different representations of the same molecule, for instance, 2D and 3D, are used as learning signals.

Improving the interpretability of the model is another critical area for development, enabling researchers to understand which molecular features most influence predictions. Finally, expanding the scope of the modelling to include dynamic chemical reactions and time-evolving processes, rather than limiting the analysis to static molecular properties, represents a promising avenue for future advancements.

In conclusion, the intersection of GNNs and LLMs offers a powerful and flexible framework for addressing complex challenges in computational chemistry. As research in this area progresses, we expect significant breakthroughs in both methodology and application, leading to more accurate, efficient, and interpretable models.

## Supplementary Material

btag387_Supplementary_Data

## Data Availability

The data underlying this article are publicly available. The GitHub repositories corresponding to each model are listed in Supplementary Table 18.
