# Supplementary material for: A survey of models composed of graph neural networks and large language models for molecular science
Source: Bioinformatics. 2026 Jun 15;42(7):btag387. doi: 10.1093/bioinformatics/btag387 (PMC13326746; doi:10.1093/bioinformatics/btag387)
Supplement: btag387_Supplementary_Data [file btag387_supplementary_data.pdf]

GNN and LLM Explanations

This appendix section provides explanations of the used GNNs (third column of Table 1) and the used LLMs (fourth column of Table 1).

Graph Neural Networks (GNN)

Several GNNs have been used in models that use both GNN and LLM (Li et al. (2024)). Nevertheless, only some of them have been used in molecular analysis models. The following enumerates only these GNNs.

1. **Graph Convolutional Network** (GCN) introduces a spectral-based convolutional operation, using a simplified Chebyshev polynomial expansion.
2. **Topology Adaptive Graph Convolutional Networks** (TAGCN) (Du et al. (2018)) is a variation of GCN, which effectively considers the various types of edge present in the graph in the message-passing mechanism.
3. **Relational Graph Convolutional Neural Network** (RGCN) (Schlichtkrull et al. (2018)) is another variation of GCN designed to adapt to handle highly multi-relational graph structures. Unlike standard GCNs that use a single transformation for all edges, RGCN perform relation-specific message passing to capture heterogeneous edge types.
4. **Graph Transformer** (GT) adapts transformer architectures to process graphs efficiently. It uses self-attention mechanisms to capture long-range dependencies without explicit locality constraints.
5. **Graph Isomorphism Network** (GIN) provides a theoretically powerful architecture that distinguishes different graph structures. This model captures multi-hop and hierarchical information in graphs.
6. **Graph Isomorphism Network with Edges** (GINE) (Hu et al. (2020a)) is a variation of GIN designed to work with edge features, which makes it more suitable for applications in molecular analysis based on graph description of compounds. Instead of simply summing up neighboring node features, GINE uses edge features in the message-passing step.
7. **Graph Diffusion Transformer** (GraphDiT) (Liu et al. (2024)) is a variation of GT trained as a diffusion model to generate molecular graphs for inverse molecular design under multiple property constraints.

Large Language Models (LLM)

Similarly to the previous subsection, we list the LLMs used in models dedicated to molecular analysis.

1. **Text-to-Text Transfer Transformer** (T5) (Raffel et al. (2019)) was developed by Google Research. The model treats all tasks as text-to-text problems, meaning that both input and output are in natural language. It performs well in a wide range of tasks, including translation, summarization, and question answering.
2. **MolT5** (Edwards et al. (2022)) is a molecular-focused chemistry adaptation of T5. It was designed for tasks such as molecular property prediction, reaction prediction, and molecule generation.
3. **Vicuna** (Chiang et al. (2023)) is an open-source LLM. It was fine-tuned on high-quality user-shared conversations, which were collected from platforms like ShareGPT. It is considered a strong alternative to ChatGPT, especially for research and open-access applications.
4. **ChatGPT** (Open AI (2023)) was developed by OpenAI, based on GPT-3.5 and GPT-4. It was fine-tuned for conversational AI, with reinforcement learning from human feedback. It is used for code generation, text completion, creative writing, and customer support.
5. **Bidirectional Encoder Representations from Transformers** (BERT) (Devlin et al. (2019)) was developed by Google AI, one of the first major transformer-based models. It uses bidirectional training, making it highly effective for tasks such as question answering and sentiment analysis. It is still widely used for applications where deep contextual understanding is needed.
6. **SciBERT** (Beltagy et al. (2019)) is a domain-specific BERT model trained on scientific texts developed by Allen Institute for AI. It was designed for scientific tasks such as paper classification, biomedical text understanding, and citation prediction. It outperforms regular BERT in scientific and technical domains.
7. **Galactica** (Taylor et al. (2022)) was developed by Meta AI, designed for scientific and technical text processing. It was trained on millions of academic papers, mathematical formulas, and code.
8. **MegaMolBART** (Irwin et al. (2022)) is a domain-specific BART model (Lewis et al. (2020)), specifically designed for tasks in cheminformatics and molecular sciences, including molecular representation learning, molecule generation, retrosynthesis prediction, and property prediction. This transformer-based deep learning model is specifically designed to operate on SMILES (Weininger (1988)) representations.
9. **LLaMA** (Touvron et al. (2023a)) is a series of open-weight LLMs created by Meta (Facebook). These high-quality models can be downloaded and run locally or on private servers (subject to licensing) and are widely used as the base for community fine-tunes (e.g., Llama-2, Llama-3, Llama-3.1). They have applications in chatbots, coding assistants, research and custom domain fine-tuning.
10. **Mistral** (Jiang et al. (2023)) is a family of LLMs developed by Mistral AI. These models are compute-efficient and deliver strong performance per parameter (e.g., Mistral-7B, Mixtral Mixture-of-Experts). They are suitable for edge deployment, efficient API usage, multilingual chat, coding and reasoning tasks.
11. **Qwen** (Bai et al. (2023)) is a family of LLMs developed by Alibaba, also known as Qwen or Tongyi Qianwen. These models support multiple languages and are available in various sizes, including Qwen-7B, Qwen-72B, and Qwen-Coder. They are used for Chinese and English assistants, coding tasks, and enterprise applications within Alibaba's ecosystem and beyond.
12. **Granite** (Mishra et al. (2024)) is a line of open models developed by IBM, designed to meet enterprise and governance requirements. These models are trained on curated datasets with a focus on trust, safety, and compliance. They are used in business chatbots, internal tools, and domain-specific assistants where data governance and traceability are critical.

General Model Architectures

This appendix section provides an overview of the general model architectures discussed in Section *Specific definition of models with GNN-LLM integration for molecule analysis*. The diagrams depict the inputs—such as molecular graphs, text, prompts, and images—entering the model and the corresponding outputs for each model and chemical task. In all the cases, *Text descriptor* does not mean the description of the molecule as SMILES or other formats but a free explanation of the molecule in natural language.

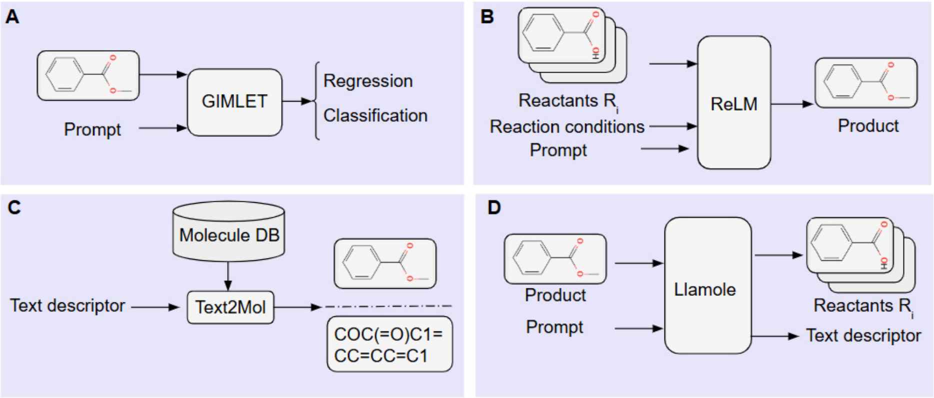

Figure 2 General model architecture of **A:** GIMLET, **B:** ReLM, **C:** Text2Mol, and **D:** Llamole.

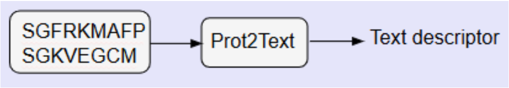

Figure 3 General Prot2Text model architecture.

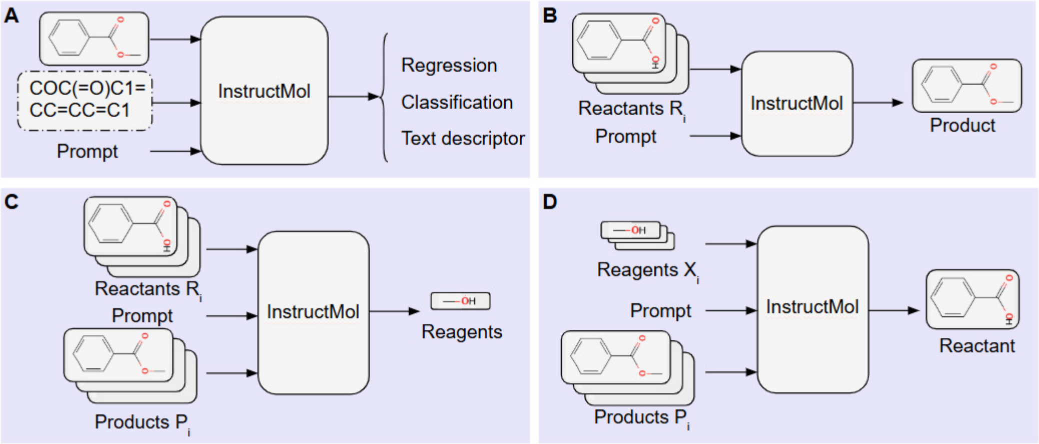

Figure 4 General model architecture for InstructMol across its different tasks; **A:** Molecular property prediction, classification, and generative molecule description, **B:** Chemical reaction prediction - Forward reaction prediction, **C:** Chemical reaction prediction - Reagent prediction and, **D:** Chemical reaction prediction - Retrosynthesis. The dashed-dotted line means that the input is optional.

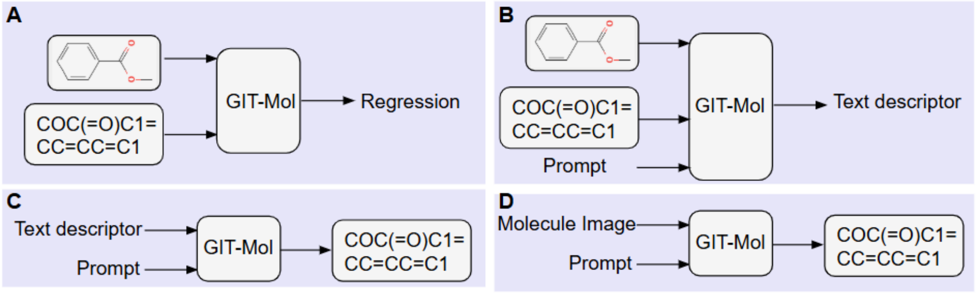

Figure 5 General model architecture of GIT-Mol across its different tasks; **A:** Molecular property prediction, **B:** Generative molecule description, **C:** Text-to-molecule generation, and **D:** Molecule image naming.

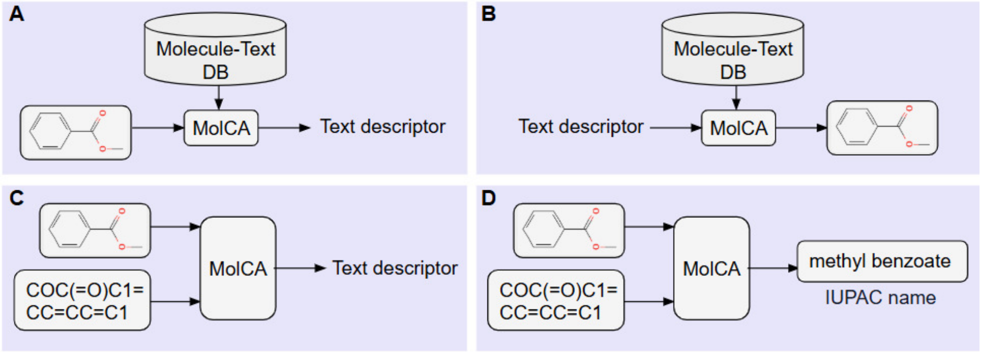

Figure 6 General model architecture of MolCA across its different tasks; **A:** Molecule-to-descriptor retrieval, **B:** Descriptor-to-molecule retrieval, **C:** Generative molecule description, and **D:** Generative molecule naming.

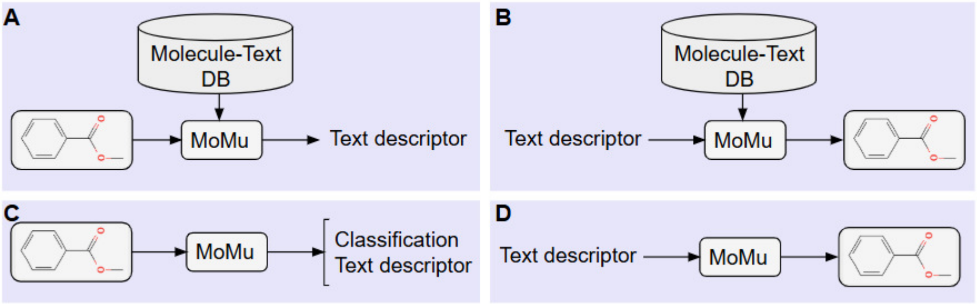

Figure 7 General model architecture of MoMu across its different tasks; **A:** Molecule-to-descriptor retrieval, **B:** Descriptor-to-molecule retrieval, **C:** Molecular property classification and Generative molecule description, and **D:** Text-to molecule generation.

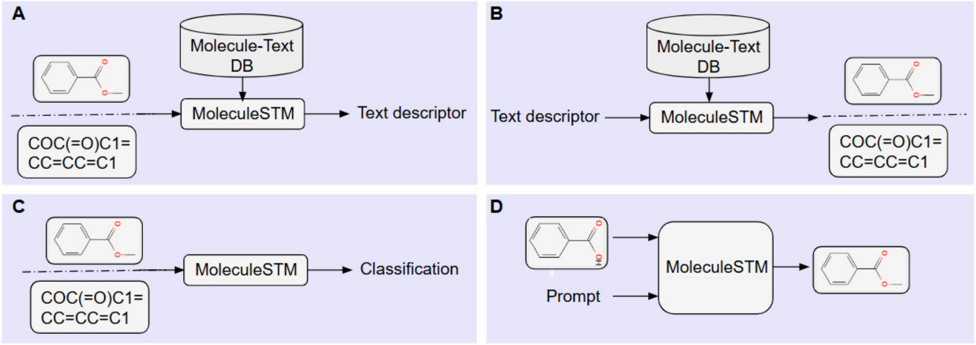

Figure 8 General model architecture of MoleculeSTM across its different tasks; **A:** Molecule-to-descriptor retrieval, **B:** Descriptor-to-molecule retrieval, **C:** Molecular property classification, and **D:** Molecule editing.

Specific Model Architectures

This appendix section presents detailed schematics for selected models and chemical tasks described in Section *Specific definition of models with GNN-LLM integration for molecule analysis*. It explains how input features and embeddings are processed, how molecular graph and textual representations are handled, and provides insight into the internal design choices of each model.

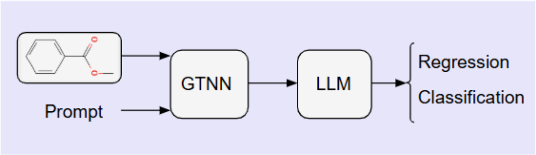

Figure 9 GIMLET detailed model architecture consists of a GTNN, as GNN, that processes the input data, followed by a T5-based LLM for molecular property prediction or classification.

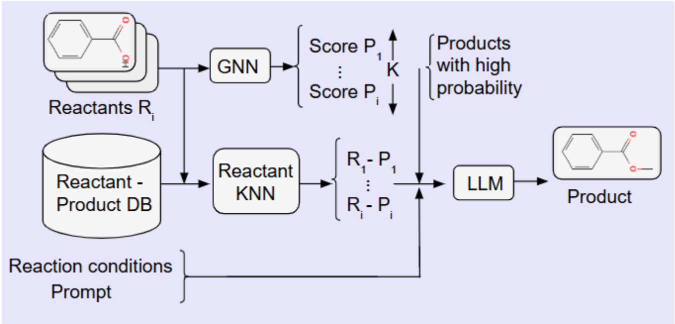

Figure 10 The ReLM model architecture for chemical reaction prediction utilizes either a GCN (LocalRetro) or TAGCN (MolR) as the GNN to generate high-probability product candidates, and subsequently uses the Vicuna or ChatGPT LLMs for the final prediction of chemical reaction products.

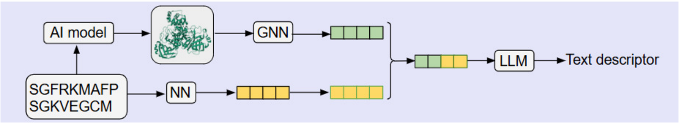

Figure 11 The Prot2Text model architecture for protein function generation. The AI Model (AlphaFold2) generates the protein structural information. An RGCN encodes the residue-level protein graph into a structural embedding that captures structural and relational features. In parallel, the amino-acid sequence is encoded using the ESM-2 transformer model as a NN, which is a protein language model, and subsequently projected to match the dimensionality of the graph embedding. This unified multimodal representation is formed by fusing these embeddings in the final encoder stage. A GPT-2 LLM decoder then uses this fused representation to generate free-text functional descriptions of the input protein.

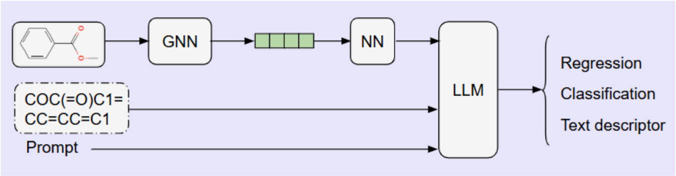

Figure 12 InstructMol model architecture for the molecular property prediction, classification, and generative molecule description tasks. The model employs the GIN from MoleculeSTM as the GNN and a Vicuna-7B as the LLM. The dashed-dotted line means that the input is optional.

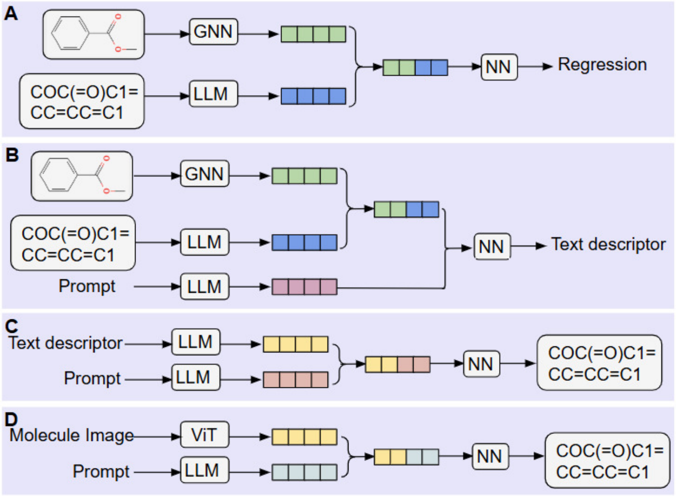

Figure 13 GIT-Mol model architectures for **A:** Molecular property prediction, **B:** Generative molecule description, **C:** Text-to-molecule generation, and **D:** Molecule image naming. In all cases, the GNN employed is the GIN model from the pre-trained MoMU model. The LLM used can either be MolT5 or SciBERT, althought SciBERT is reported to be specifically used for task B. The molecule image is processed using a Vision Transform. The NN used is a MLP. The curly bracket with an arrow denotes the arcitecture of the GIT-Former.

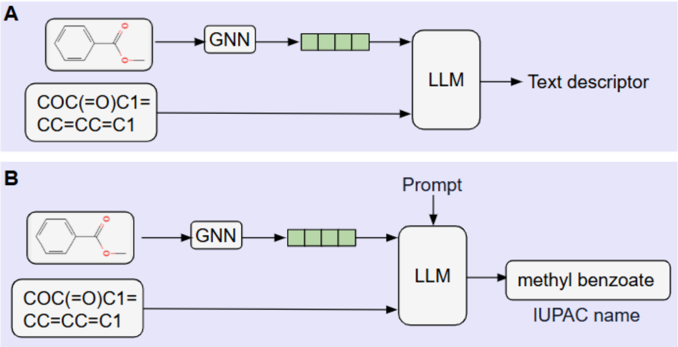

Figure 14 MolCA model architecture across its different tasks; **A:** Generative molecule description, and **B:** Generative molecule naming. In both cases, the GNN employed is a GINE. For the LLM task, Galactica is used; however, for task A, SciBERT can also be utilized as an alternative LLM. In task B, the prompt is generated by the model, and it is not provided by the user, in order to enhance the generation of an IUPAC name.

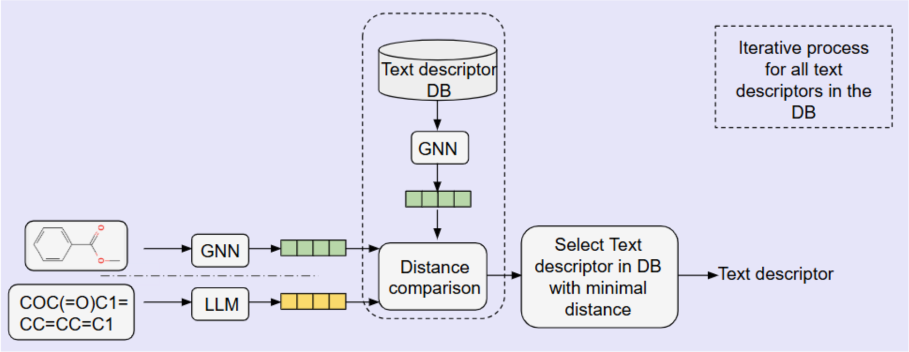

Figure 15 Text2Mol model architecture uses a GCN as the GNN and a SciBERT as the LLM. The molecule database (DB) is also provided as input. For each molecule in the database, distance comparison is performed using a modified cross-modal attention model. Further details about the cross-modal attention model can be found in either Section *Text2Mol* or (Edwards et al. (2021)).

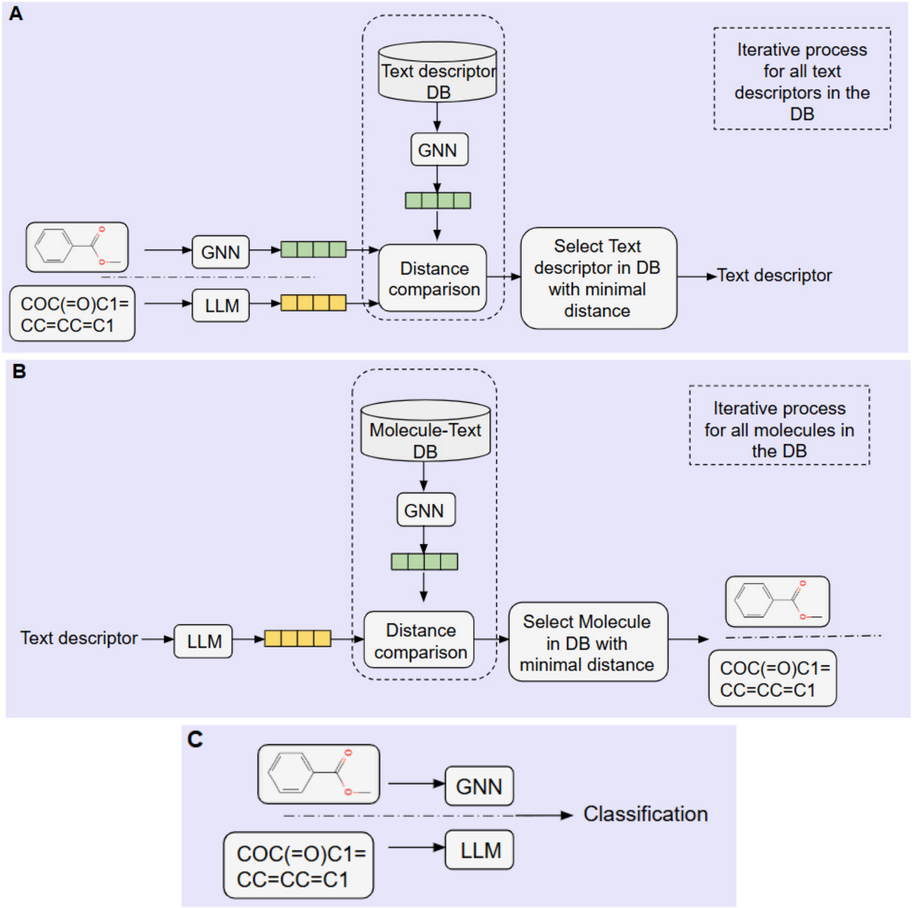

Figure 16 MoleculeSTM model architecture across its different tasks; **A:** Molecule-to-descriptor retrieval, **B:** Descriptor-to-molecule retrieval, **C:** Molecular property classification. The dashed-dotted line means that the input is optional. The model uses a GIN as the GNN. In MoleculeSTM, the LLMs BERT and MegaMolBART can be used either simultaneously or individually. In task C, the model directly classifies the molecular properties of the input data (either the molecular 2D graph or the SMILES representation) using either the GNN or the LLM, with no other model architectures or GNNs/LLMs used afterward.

Comparative Evaluation Framework: Databases, Metrics, Baselines, and Published Results

	Model	GNN	LLM	Task	Database	Metrics	Baseline models
1	GIMLET	GT	T5	Molecular property prediction	ESOL (Delaney (2004)), Lipophilicity (Wu et al. (2018)), FreeSolv (Mobley and Guthrie (2014)), ChEMBL (Zdrazil et al. (2024))	RMSE	KV-PLM (Zeng et al. (2022)), MoMu,GCN, GAT (Veličković et al. (2017)), GIN,Graphormer (Ying et al. (2021)), Galactica
2	GIMLET	GT	T5	Molecular property classification	BACE (Subramanian et al. (2016)), HIV (Wu et al. (2018)), MUV (Gardiner et al. (2011)), Tox21 (Wu et al. (2018)), ToxCast (Richard et al. (2016)), BBBP (Martins et al. (2012)), CYP450 (Preissner et al. (2010)), ChEMBL, PCBA (Wang et al. (2011))	ROC-AUC	KV-PLM, MoMu, GCN, GAT, GIN, Graphormer
3	ReLM	GCN, TAGCN	GPT-3.5, Vicuna	Chemical reaction prediction	Imidazo (Shi et al. (2023)), NiCOlit (Shi et al. (2023)), Rexgen-30k (Coley et al. (2019)), Rexgen-40k	HIT@K	MolR (Wang et al. (2022a)), LocalRetro (Chen and Jung (2021))
4	Llamole	GIN, GraphDiT	LLaMA, Mistral, Qwen, Granite, T5	Chemical reaction prediction	PubChem (Kim et al. (2022)), MoleculeNet (Wu et al. (2018)), ChEMBL, ZINC (Irwin et al. (2012)), PI1M (Ma and Luo (2020)), MSA (Thornton et al. (2012)), USPTO (Fang et al. (2023))	ROUGE, BLEUR, Validity, Similarity, MAE	GraphGA (Gao et al. (2022))
5	Prot2Text	RGCN	ChatGPT	Generative molecule descriptor	SwissProt	BLEU, ROUGE-1, ROUGE-2, ROUGE-L, BERT	Graph Transformer, RGCN, GPT-2
6	InstructMol	GIN	Vicuna-7B	Molecular property prediction	QM9 (Ramakrishnan et al. (2014))	MAE	Alpaca (Dubois et al. (2024)), Baize (Xu et al. (2023)),Galactica, LLaMA (Touvron et al. (2023b)), Vicuna, Mol-instruction (Fang et al. (2023))
7	InstructMol	GIN	Vicuna-7B	Molecular property classificatiom	BACE, BBBP, HIV	ROC-AUC	ChemBERTa (Ahmad et al. (2022)), DMP (Zhu et al. (2023)),KV-PLM, GraphCL (You et al. (2020), GraphMVP-C (Liu et al. (2022)), MoMu,MolFM (Luo et al. (2023)), Uni-Mol (Zhou et al. (2023)), Galactica, Vicuna,LLaMA, MolCA
8	InstructMol	GIN	Vicuna-7B	Chemical reaction prediction	Mol-instruction	FTS[1], BLEU (Papineni et al. (2002)), Exact Match, Levensthtein distance	Alpaca, Baize, ChatGLM (Zeng et al. (2023)), LLaMA, Mol-instruction, Vicuna, Text+ChemT5 (Christofidellis et al. (2023)), Molecular transformer (Schwaller et al. (2019)), Retroformer-untyped (Wan et al. (2022))
9	InstructMol	GIN	Vicuna-7B	Generative molecule description	PubChem, ChEBI-20 (Edwards et al. (2021)), USPTO, MoleculeNet	BLEU-2, BLEU-4, ROUGE-1, ROUGE-2, ROUGE-L,METEOR (Banerjee and Lavie (2005))	MolT5-base, MoMu,MolFM, MolXPT (Liu et al. (2023)),GIT-Mol, MolCA, Text+ChemT5, GPT-3.5, GPT-4-0314, BioMedGPT-10B (Luo et al. (2024)), Mol-Instruction
10	GIT-Mol	GIN	MolT5	Molecular property classification	Sider (Kuhn et al. (2015)), ClinTox (Novick et al. (2013)), BACE, Tox21, ToxCast, BBBP	AUC	KV-PLM, GraphCL, GraphMVP, MoMu, Mole-BERT
11	GIT-Mol	GIN	SciBERT, MolT5	Generative molecule description	PubChem, ChEBI-20	BLEU-2, BLEU-4, ROUGE-1, ROUGE-2,ROUGE-L, METEOR	SciBERT, MolT5

	Model	GNN	LLM	Task	Database	Metrics	Baseline models
12	GIT-Mol	-	MolT5	Text-to-molecule generation	ChEBI-20	FTS, BLEUR, Exact Match, Levenshtein distance, validity	SciBERT, MolT5
13	GIT-Mol	ViT[2]	MolT5	Molecule image naming	ChEBI-20	FTS, BLEUR, Exact Match, Levenshtein distance, validity	SwinOCSR (Xu et al. (2022))
14,	MolCA	GINE	Galactica	Molecule-to-Descriptor retrieval, Descriptor-to-molecule retrieval	PubChem324k, PCDes (Zeng et al. (2022)), MoMu	Accuracy, Recall@20	MoleculeSTM, KV-PLM,SciBERT, MoMu
16	MolCA	GINE	Galactica, MolT5	Generative molecular description	PubChem324k, ChEBI-20	BLEU-1, BLEU-4, ROUGE-1, ROUGE-2, ROUGE-L, METEOR	T5, MolT5, MoMu
17	MolCA	GINE	Galactica	Generative molecule naming	PubChem324k	BLEU, ROUGE,METEOR	MolT5
18	Text2Mol	GCN	SciBERT	Descriptor-to-molecule retrieval	ChEBI-20	MRR, HIT@1, HIT@10	MLP, GCN
19,	MoMu	GIN	SciBERT, KV-PLM	Molecule-to-Descriptor retrieval, Descriptor-to-molecule retrieval	PCDes	HIT@1, Recall@20	SciBERT, KV-PLM
21	MoMu	GIN	SciBERT, KV-PLM	Molecular property classification	BBBP, Tox21, ToxCast, SIDER, ClinTox, MUV, HIV, BACE	ROC-AUC, STD	Infomax (Veličković et al. (2019)), EdgePred (Hamilton et al. (2017)), AttrMasking (Hu et al. (2020b)) ,GraphCL, ContextPred (Hu et al. (2020b))
22	MoMu	GIN	SciBERT, KV-PLM	Generative molecule descriptor	ChEBI-20	BLEU-2, BLUE-4,ROUGE-L, METEOR, Text2Mol	MolT5
23	MoMu	GIN	SciBERT, KV-PLM	Text-to-molecule generation	ZINC250K	Visual comparison	MolT5
24,	MoleculeSTM	GIN	BERT, MegaMolBART	Molecule-to-Descriptor retrieval, Descriptor-to-molecule retrieval	DrugBank (Wishart et al. (2017))	Accuracy	KV-PLM
26	MoleculeSTM	GIN	BERT, MegaMolBART	Molecular property classification	BACE, HIV, MUV, Tox21, ToxCast, BBBP, ClinTox, PCBA, Sider	ROC-AUC	MegaMolBART, KV-PLM, AttrMask, GraphMVP,ContextPred, InfoGraph (Sun et al. (2020)), MolCLR (Wang et al. (2022b))
27	MoleculeSTM	GIN	BERT, MegaMolBART	Molecule editing	ZINC	HIT@Ratio, visual analysis	Random, PCA, GS, High-variance

Table 3: Summary of the models and their tasks, along with the databases, metrics, and baseline models used for evaluation. Downstream tasks numbers 1 to 27 from Table 2. FTS[1]: Fingerprint Tanimoto Similarity. ViT[2]: Vision Transform, an image NN, is listed under the GNN column to avoid the creation of an additional column.

Ten different models, presented in the last five years, have been depicted and classified into three different classes for the first time. Moreover, a deep analysis on their application has been presented, showing some specific details of the GNNs and LLMs used. The aim has been to introduce these models to better understand the state of the art in this field. Thus, the focus of the paper was to present a clear classification and description for researchers, who can download the code and use it with more knowledge of the models and applications.

To facilitate a comprehensive understanding of the models, Table 3 show a recompilation of the databases used to test the models, the evaluation metrics, and the models they have compared to. Furthermore, to provide a general overview of the models, we also include their GNN and LLM, although also described in Table 1. The first column of Table 3 corresponds to the tasks identifiers presented in Table 2. The Github link to each model is provided in Table 18.

Analysis, prediction, and discovery of chemical compounds have become a trending topic in molecular science as a result of the advent of mathematical models that achieve impressive results, defined as accuracy of classification or regression, or validity in the generated new compounds. The models that integrate GNNs and LLMs applied to several downstream tasks have been the cornerstone of these achievements.

It is also important to consider the diversity of the datasets used in these models. As shown in Table 3, a wide range of molecular databases are used, reflecting different objectives and chemical domains. Although most of the models discussed in this article focus primarily on small-molecule tasks, the authors also highlight applications involving broader molecular classes such as polymers or proteins. Llamole is a great example: by modifying the training datasets, the model can shift its molecular objective, from drug-like molecules to polymers, without altering the underlying architecture. This represents a significant step toward more adaptable and generalizable molecular foundation models. Future work could extend this adaptability to additional molecular classes, such as macromolecules, metal–organic frameworks, or advanced materials.

Table 4 to Table 16 present detailed experimental results compiled from various studies, with each table corresponding to a distinct category of chemical prediction or generation task, such as molecule-to-descriptor retrieval, molecular property classification, or generative molecule description. With the aim of the results being as much comparable as possible, the results in each table have been extracted from the same source of information.

This task-specific organization allows for clearer comparison of model performance within each task while also facilitating a comprehensive understanding of the strengths and limitations of each approach in its designated context. The tables represent a distilled view of the results reported in the original publications. As this article focuses primarily on the analysis of models rather than databases, when identical metrics are reported across different models for the same databases, we have computed the mean of the reported values for each database. This approach ensures a fair and consistent comparison among the models. Furthermore, ablation studies demonstrating that the complete model outperforms its variants are not included.

The experimental results summarized from Table 4 to Table 16 provide quantitative evidence of the performance gains enabled by the integration of LLMs and GNNs across different chemical tasks, as well as a comparison between the models for each task.

Table 4 and Table 5 collectively evaluate bidirectional retrieval tasks - molecule-to-descriptor and descriptor-to-molecular - in PubChem324k and ChEBI-20 datasets. Across both tasks, the MolCA model consistently demonstrates higher accuracy and Recall@20, with 66.6% and 94.6% in the molecule-to-descriptor task and 66.0% and 93.5% in the reverse direction, respectively. Furthermore, both MoMu and MolecueleSTM-G show moderate and consistent performance in the two tasks. Text2Mol achieved a higher Recall@10 of 81.1% compared to the GCN2 results. Although Text2Mol cannot be compared against the other models, the similarity of the Recal@K metric, allow us an insight into the top-k retrieval capabilities.

MolCA also has the best overall performance on the generative molecule description task (Table 6), outperforming three other GNN-LLM models in all BLEU, ROUGE, and METEOR metrics. MoMu also performs competitively, particularly in ROUGE-L and METEOR (both being 0.577), indicating strong semantic alignment. InstructMol-GS shows moderate and balanced results across metrics, while GIT-Mol, despite lower BLEU scores, performs comparably on ROUGE and METEOR values, suggesting strengths in content recall but reduced lexical precision.

Prot2Text results are not directly comparable with the three generative molecule description models discussed earlier, as they are evaluated on a different dataset. Nevertheless, Prot2Text provides a clear demonstration of the benefits of combining GNN and LLM within a multimodal generative framework. As shown in Table 7, Prot2Text achieves the highest performance in all BLEU, ROUGE, and BERTS score metrics. Table 7 compares Prot2Text with two ablation-style baselines: one that uses only a NN with an LLM (vanilla-Transformer + GPT-2) and another that uses a unimodal architecture of a GNN paired with an LLM without a NN (RGCN + GPT-2).

Table 8 reports results for molecular prediction tasks, where performance is measured using mean absolute error (MAE) or root mean square error (RMSE), depending on the dataset. Due to the use of different metrics across tasks, the two GNN-LLM models included - InstructMol and GIMLET- are not directly compared to each other. Instead, each is evaluated against a baseline model representing either a LLM model (Galactica) or GNN model (Graphormer). In the QM9 dataset, where MAE is the evaluation metric, InstructMol achieves a remarkable low MAE of 0.005, surpassing Galactica, which reports an MAE of 0.568. For datasets where RMSE is used (ESOL, Lipophilicity, and FreeSol), Graphormer demonstrates a lower average RMSE of 1.284 compared to GIMLET's RMSE of 2.527. This performance gap should be interpreted in light of the zero-shot evaluation setting used by GIMLET. In particular, predicting unseen physicochemical properties using only natural-language supervision is difficult due to the continuous and high-dimensional output space. While this does not necessarily lead to state-of-the-art RMSE values compared to fully supervised GNN models, it highlights the potential of GNN–LLM architectures for numerically grounded, instruction-based molecular reasoning in low- or zero-shot settings.

The molecular property classification results presented in Table 9 indicate that GIT-Mol achieves the highest average ROC-AUC score of 77.49. This is followed by the MoleculeSTM variants, with STM-G obtaining a score of 75.89 and STM-S slightly higher at 76.59. The Instruct-GS and MoMu variants yielded slightly lower performance, with scores ranging from 74.37 to 75.63. In contrast, GIMLET was the lowest performing one at 65.08.

Model	Accuracy	Recall@20
MolCA	66.6	94.6
MoMu-S	40.9	86.2
MoMu-K	41.8	87.5
MoleculeSTM-G	45.8	88.4

Table 4 Accuracy and Recall@20 results for molecule-to-descriptor prediction on PubChem324k datasets. MoMu variants differ by the LLM used: S for SciBERT and K for KV-PLM. Results extracted from (Liu et al. (2023b)).

Model	Accuracy	Recall@10	Recall@20
MolCA	66.0	-	93.5
MoMu-S	40.8	-	86.1
MoMu-K	41.6	-	87.8
MoleculeSTM-G	44.3	-	90.6
Text2Mol	-	81.1	-
GCN2	-	68.9	-

Table 5 Descriptor-to-molecule results across the ChEBI-20 (Recall@10) and PubChem324k (accuracy and Recall@20) dataset. A dash (–) indicates that results are not reported. MoMu variants differ by the LLM used: S for SciBERT and K for KV-PLM.

Model	BLEU-2	BLEU-4	ROUGE-1	ROUGE-2	ROUGE-L	METEOR
InstructMol-GS	0.475	0.371	0.566	0.394	0.502	0.509
GIT-Mol*	0.352	0.263	0.575	0.485	0.560	0.533
MolCA	0.620	0.531	0.681	0.537	0.618	0.651
MoMu	0.560	0.474	-	-	0.577	0.577

Table 6 Generative molecule description results using the ChEBI-20 dataset. *Trainied and tested using a combination of ChEBI-20 and PubChem datasets. A dash(–) indicates no result reported.

Model	BLEU	ROUGE-1	ROUGE-2	ROUGE-L	BERT
Prot2Text	0.351	0.506	0.427	0.485	0.843
RGCN + GPT-2	0.216	0.362	0.280	0.344	0.789
Vanilla Transformer + GPT-2	0.158	0.278	0.194	0.261	0.756

Table 7 Published results from Pro2TXT for generative molecule description results using the SwissProt dataset.

The results of the chemical reaction prediction task, shown separately for ReLM (Table 10), InstructMol (Table 11) and Llamole (Table 12), highlight distinct strengths across the three models. ReLM excels in out-of-distribution forward reaction prediction, achieving a high accuracy value of 0.914 (K=3) and 0.87 (K=4) when combining MolR with Vicuna, outperforming the standalone GNN MolR baseline of 0.513 (K=3 and K=4). In contrast, InstructMol is evaluated in forward prediction, retrosynthesis, and reagent prediction at the same time, where InstructMol-GS shows strong performance with higher exact match (0.357) and BLEU (0.839) scores, along with higher FTSs and 100% validity. Llamole complements these models by focusing on multimodal, constraint-based inverse molecular design and retrosynthetic reasoning. It achieves high chemical validity (0.913) and similarity to target structures (0.142), while also generating coherent retrosynthesis descriptions, with higher BLEU-4 (0.254) and ROUGE-L (0.427) scores than in-context baselines (0.030 and 0.141, respectively). For reaction prediction, Llamole reaches a BA of 0.623 for drug molecules and a lower error in polymer property prediction (MAE = 0.653), outperforming both the non-LLM baseline GraphGA (BA of 0.536 and MAE of 0.847) and in-context LLM prompting (BA of 0.051 and MAE of 5.463). Overall ReLM excels in constrained candidate selection, InstructMol offers broader prediction capabilities and greater chemical correctness, and Llamole demonstrates strong multimodal integration for structure generation and retrosynthetitc planning. Future works evaluating all three models under a unified benchmark would offer deeper insights.

The results of molecule editing (Table 13) show that the baseline methods achieve lower satisfactory hit ratios, with Random at 24.6 and GS at 39.6. In contrast, MoleculeSTM variants perform substantially better, with both variants achieving 64.1. This highlights that MoleculeSTM's SMILES and graph-based encoders enhance semantic understanding of natural language, enabling more effective generation of molecules with desired properties. The satisfactory hit ratio is a metric, which is the difference between the output molecule and the combination of the input molecule and its prompt over a threshold, in this case it was set to 0. More details of this metric can be found at Liu et al. (2023a).

Tables 14 and 15 compare the performance of MolCA and GIT-Mol, respectively, highlighting the strength of each model in molecule naming tasks. MolCA excels in text-based generative naming, outperforming MolT5 with the highest BLEU, ROUGE, and METEOR scores (e.g., BLEU-4: 66.6, METEOR: 72.1). GIT-Mol leads in image-to-name generation, achieving high scores in all metrics, including a BLEU of 0.924 and fingerprint similarities greater than 0.89, indicating strong structural accuracy. Together, the results showcase the complementary strengths of GNN-LLMs in different naming modalities.

The text-to-molecule generation task is addressed by two models, MoMu and Git-Mol. In the MoMu study, evaluation is performed qualitatively through a visual comparison with MolT5, rather than using quantitative metrics. A key strength of MoMu's lies in its ability to generate multiple, diverse, and interpretable molecular structures for abstract or subjective prompts (e.g., "the molecule is beautiful"), while MolT5 struggles with such inputs due to its reliance on explicit structural descriptions. In contrast, Git-Mol (Table 16) shows performance comparable to two LLM baselines (SciBERT and MolT5-base), while achieving a higher proportion of valid molecules and maintaining high molecular similarity.

These results highlight the synergistic benefits of combining GNNs with LLMs in a variety of molecule-related tasks. While Tables 4, 5, 6, and 9 present comparisons among the different GNN-LLM models analyzed in this study, in their respective original publications, these models are also evaluated against stand-alone GNN or LLM baselines. The findings reported in those works are consistent with the results shown in Tables 7, 8, 10 to 16, where the combination of GNNs with LLMs performs on a par with or exceeds its standalone GNN or LLM counterparts.

Method	MAE	RMSE
Galactica	0.568	–
InstructMol	0.005	–
Graphormer	–	1.284
GIMLET	–	2.527

Table 8 Molecular property prediction results. MAE is used for QM9, and RMSE is the average between the results published in GIMLET for the databases ESOL, Lipophilicity, and FreeSolv. A dash (–) indicates no result reported.

Model	ROC-AUC
Instruct-G	75.63
Instruct-GS	74.47
GIMLET	65.08
MoMu -S	74.37
MoMu -K	74.47
MoleculeSTM - S	76.59
MoleculeSTM - G	75.89
GIT-Mol (G+S)*	77.49

Table 9 Molecular property classification results. Reported values are the average ROC-AUC scores across the BACE, BBBP, and HIV datasets. Model variants are denoted as S (SMILES-based), G (2D graph-based), and GS (graph and SELFIES-based). MoMu variants differ by the LLM used: S for SciBERT and K for KV-PLM. *GIT-Mol average is computed from two available datasets (BBBP and BACE)

.

	Accuracy	
Model	K=3	K=4
MolR	0.513	0.513
ReLM (MolR + Vicuna)	0.914	0.87
ReLM (MolR + GPT-3.5)	0.865	0.815

Table 10 Accuracy of the ReLM model on out-of-distribution settings for forward chemical reaction prediction. Values represent the average accuracy reported in the original study across four datasets: Imidazo, NiCOlit, Rexgen-30k, and Rexgen-40k. K=3 and K=4 indicate the number of candidate products generated by the GNN and passed to the LLM.

The decision regarding the LLM's configuration is also important. Some LLMs allow fine-tuning, meaning they can be retrained to improve performance, while others are frozen and can only be used as they are. Experimentation has shown that LLMs that can be fine-tuned typically achieve better results; however, they come with the challenge of significant computational effort required for training or retraining. The choice of whether to use a fine-tunable model or a frozen one ultimately depends on the specific application and available resources.

We explored, analyzed, and demonstrated ten distinct molecular tasks, ranging from molecule-to-descriptor retrieval to molecule image naming. Yet, the models' potential extends far further. Llamole could support generative molecule description, property prediction, classification, or text-to-molecule generation. Text2Mol could enhance molecule-to-descriptor retrieval, and ReLM could enable molecule editing. These tasks do not include the full breadth of what is possible in computational chemistry. The scope of this article was intentionally aligned with the capabilities of the models under study, but many additional tasks exist. Beyond the tasks studied, one could generate molecules from text while simultaneously predicting their properties or assigning them to a specific class, or design multi-conditional prompts to guide molecular generation under multiple constraints. Future work will likely focus on the development of new models that integrate GNNs and LLMs to tackle an even wider array of chemical tasks.

Model	Exact	BLEU	Levenshtein	RDK FTS	MACCS FTS	Morgan FTS	Validity
Vicuna	0.000	0.041	38.838	0.023	0.016	0.009	0.028
Mol-Instruction	0.033	0.528	27.219	0.278	0.453	0.235	1.000
InstructMol-G	0.112	0.794	22.053	0.470	0.644	0.389	1.000
InstructMol-GS	0.357	0.839	14.827	0.658	0.756	0.618	1.000

Table 11 Average performance of the InstructMol model across three chemical reaction prediction tasks (Reagent Prediction, Forward Reaction Prediction, Retrosynthesis) using the Mol-instruction database. Model variants are denoted as G (2D graph-based) and GS (graph and SELFIES-based).

Method	Validity	Similarity	BLEU-4	ROUGE-L	BA* (Drug)	MAE (Polymer)
GraphGA	0.885	0.112	-	-	0.536	0.847
In-Context Learning	0.167	0.024	0.030	0.141	0.051	5.463
Llamole	0.913	0.142	0.254	0.427	0.623	0.653

Table 12 Chemical reaction prediction for rethrosynthesis performance of LLamole for both molecular (drug) and polymer compounds. *BA is the Balanced Accuracy. A dash (–) indicates no result reported. The In-Context Learning model is Llama-2-7B.

It is evident that LLMs and GNNs will play an increasingly important role together in future molecular science applications, particularly for tasks that involve natural-language instructions or textual specifications. In such settings, integrating LLMs with GNNs can substantially enhance flexibility and usability. At the same time, GNN-based solutions alone remain highly effective for many problems where structured molecular representations and numerical objectives are sufficient. Consequently, the choice between GNN-only and GNN–LLM approaches should be driven by the specific requirements of the application.

Finally, to specify our evaluation, Table 17 presents a comprehensive summary of the strengths and weaknesses of the ten analyzed models: GIMLET, ReLM, Prot2Text, Llamole, InstructMol, GIT-Mol, MolCA, Text2Mol, MoMu and MoleculeSTM. Both, in terms of task coverage and architectural flexibility, the models exhibit a range of trade-offs. For example, models like GIT-Mol and MolCA demonstrate strong performance across both predictive and generative tasks, while MolCA excels in tasks involving descriptor translation and molecule generation.

Method	Satisfactory Hit Ratio
Random	24.6
PCA	29.6
High-variance	34.6
GS	39.6
MoleculeSTM-S	64.1
MoleculeSTM-G	69.1

Table 13 Molecule editing results. Average Satisfactory Hit Ratio across four task types: single-objective, multi-objective, ChEMBL binding-affinity-based, and drug relevance. Model variants are denoted as S (SMILES-based) and G (2D graph-based). GS means generic search.

Model	BLEU-2	BLEU-4	ROUGE-1	ROUGE-2	ROUGE-L	METEOR
MolT5	59.4	49.7	55.9	33.3	49.1	58.5
MolCA	75.0	66.6	69.6	48.2	63.4	72.1

Table 14 Generative molecule naming results using PubChem324k.

Model	BLEU	Exact	Levenshtein	MACCS FTS	RDK FTS	Morgan FTS	Validity
SwinOCSR	0.892	0.376	9.157	0.945	0.872	0.846	0.827
GIT-Mol	0.924	0.461	6.575	0.962	0.906	0.894	0.899

Table 15 Molecule image naming results using ChEBI-20.

Model	BLEU	Exact	Levenshtein	MACCS FTS	RDK FTS	Morgan FTS	Validity
SciBERT	0.459	0.005	55.459	0.499	0.344	0.254	0.915
MolT5-base	0.769	0.081	24.458	0.721	0.588	0.529	0.772
GIT-Mol	0.756	0.051	26.315	0.738	0.582	0.519	0.928

Table 16 Text to Molecule generation results using ChEBI-20.

Model	Strengths	Weaknesses
GIMLET	Powerful representation of molecules through graph transformers that support features on both nodes and edges.<br>T5 can be trained.	Only applicable to molecular property prediction or classification. No generative tasks reported.<br>Weaker performance against other GNN-LLM models.
ReLM	Vicuna can be trained.<br>Requires low computational resources since ChatGPT is already pretrained.<br>Best performance in chemical reaction prediction task.	Bond types are not considered on edges.<br>ChatGPT cannot be fine-tuned on specific molecules.<br>Only applicable to chemical reaction prediction.
Prot2Text	Multimodal architecture that jointly integrates protein sequence and 3D structural information using both GNNs and LLMs. The protein structural information is computed by AlphaFold2 developed by DeepMind (Google). Therefore, structural information is not part of the input.<br>Generates free-text protein function descriptions rather than fixed labels, enabling richer and more interpretable outputs.<br>Demonstrates clear performance gains when combining GNN and NN encoders with an LLM decoder, outperforming unimodal baselines.	Results are dataset-specific and not directly comparable to molecule-focused generative models.
Llamole	Multimodal modal that integrates text and molecular graph modalities for joint generation.<br>Supports controllable molecular design with property constraints.<br>Capable of multi-step rethrosyntesis planning for generated molecules.<br>Demonstrates superior performance compared to baselines across multiple metrics.	Limited scope of molecular properties considered.<br>Retrosynthesis success rate is not yet fully reliable; some generated pathways may be chemically unrealistic.
InstructMol	Vicuna can be efficiently trained, although the "GNN-LLM include" architecture needs high computational power.<br>Aligns molecular graphs and chemical sequential modalities with humans' natural language.<br>Best performance in molecular property prediction, and the three chemical reaction prediction tasks.	GIN does not incorporate edge features, so bond types are not considered. Even though some edge information can be incorporated into node attributes, some properties – such as bond types or bond distance – cannot be directly represented.
GIT-Mol	SciBERT and MolT5 can be trained.<br>Versatile — applicable to both molecular property and reaction prediction tasks.<br>Demonstrates good performance on generative tasks.<br>Best performance in molecular property classification, text-to-molecule generation, and molecule image naming tasks.	GIN does not incorporate edge features. Even though some edge information can be incorporated into node attributes, some properties – such as bond types or bond distance – cannot be directly represented.<br>Training is time-consuming.
MolCA	Since GINE incorporates features on the edges, the type of bond is considered on the architecture.<br>Galactica and MolT5 can be trained.<br>Nicely applied to molecular prediction and also generative tasks.<br>Best performance in molecule-to-descriptor, descriptor-to-molecule, generative molecule description, and generative molecule naming tasks.	The "GNN-LLM include" architecture might be very time consuming since both, GNN and LLM have to be trained at the same time. There is need of high computational power.
Text2Mol	SciBERT can be trained increasing the quality of results.<br>Not high computational requirements. High performance on molecule retrieval	GCN is one of the first GNN and they have been outperformed by GINE or GT. Moreover, the type of bond is not considered on the edges.<br>Prompts are not allowed, which decreases task specification.<br>Only applied to retrieval of molecule descriptors.
MoMu	BERT can be trained.<br>It might be the current most versatile architecture due to its generative abilities.<br>Best performance in text-to-molecule generation task.	GIN does not incorporate features on the edges then, the type of bond is not considered.<br>Prompts are not allowed, which decreases task specification.
Molecule STM	BERT and MegaMolBART can be trained.<br>The only model that presents molecule editing.<br>Best performance in molecule editing task.	GIN does not incorporate features on the edges then, the type of bond is not considered.

Table 17 Strengths and weaknesses of the analyzed models.

Model	Link
GIMLET	https://github.com/zhao-ht/GIMLET
ReLM	https://github.com/syr-cn/ReLM
Prot2Text	https://github.com/hadi-abdine/Prot2Text
Llamole	https://github.com/liugangcode/Llamole
InstructMol	https://github.com/IDEA-XL/InstructMol
GIT-Mol	https://github.com/AI-HPC-Research-Team/GIT-Mol
MolCA	https://github.com/acharkq/MolCA
Text2Mol	https://github.com/cnedwards/text2mol
MoMu	https://github.com/BingSu12/MoMu
MoleculeSTM	https://github.com/chao1224/MoleculeSTM

Table 18 Hyperlinks of the Python code of the ten GNN-LLM models.

Appendix References

W. Ahmad, E. Simon, S. Chithrananda, G. Grand, and B. Ramsundar. Chemberta-2: Towards chemical foundation models. *ArXiv*, abs/2209.01712, 2022.

J. Bai, S. Bai, Y. Chu, and et al. Qwen technical report, 2023.

S. Banerjee and A. Lavie. METEOR: An automatic metric for MT evaluation with improved correlation with human judgments. In J. Goldstein, A. Lavie, C.-Y. Lin, and C. Voss, editors, *Proceedings of the ACL Workshop on Intrinsic and Extrinsic Evaluation Measures for Machine Translation and/or Summarization*, pages 65–72, 2005.

I. Beltagy, K. Lo, and A. Cohan. SciBERT: A pretrained language model for scientific text. In *Proceedings of the 2019 Conference on Empirical Methods in Natural Language Processing and the 9th International Joint Conference on Natural Language Processing (EMNLP-IJCNLP)*, pages 3615–3620, 2019.

S. Chen and Y. Jung. Deep retrosynthetic reaction prediction using local reactivity and global attention. *JACS Au*, 1(10): 1612–1620, 2021.

W.-L. Chiang, Z. Li, Z. Lin, Y. Sheng, Z. Wu, H. Zhang, L. Zheng, S. Zhuang, Y. Zhuang, J. E. Gonzalez, I. Stoica, and E. P. Xing. Vicuna: An open-source chatbot impressing gpt-4 with 90% chatgpt quality, 2023. URL https://lmsys.org/blog/2023-03-30-vicuna/.

C. W. Coley, W. Jin, L. Rogers, T. F. Jamison, T. S. Jaakkola, W. H. Green, R. Barzilay, and K. F. Jensen. A graph-convolutional neural network model for the prediction of chemical reactivity. *Chemical Science*, 10:370–377, 2019.

J. S. Delaney. ESOL: Estimating aqueous solubility directly from molecular structure. *Journal of Chemical Information and Computer Sciences*, 44(3):1000–1005, 2004. ISSN 0095-2338.

J. Devlin, M. Chang, K. Lee, and K. Toutanova. BERT: pre-training of deep bidirectional transformers for language understanding. In *Proceedings of the 2019 Conference of the North American Chapter of the Association for Computational Linguistics: Human Language Technologies, NAACL-HLT*, volume 1, pages 4171–4186, 2019.

J. Du, S. Zhang, G. Wu, J. M. F. Moura, and S. Kar. Topology adaptive graph convolutional networks, 2018.

Y. Dubois, X. Li, R. Taori, T. Zhang, I. Gulrajani, J. Ba, C. Guestrin, P. Liang, and T. B. Hashimoto. AlpacaFarm: A simulation framework for methods that learn from human feedback. *ArXiv*, 2024.

C. Edwards, T. Lai, K. Ros, and et al. Translation between molecules and natural language. In *Proceedings of the 2022 Conference on Empirical Methods in Natural Language Processing*, pages 375–413, 2022.

Y. Fang, X. Liang, N. Zhang, K. Liu, R. Huang, Z. Chen, X. Fan, and H. Chen. Mol-Instructions: A large-scale biomolecular instruction dataset for large language models. *ArXiv*, abs/2306.08018, 2023.

W. Gao, T. Fu, J. Sun, and C. W. Coley. Sample efficiency matters: A benchmark for practical molecular optimization. In S. Koyejo, S. Mohamed, A. Agarwal, D. Belgrave, K. Cho, and A. Oh, editors, *Advances in Neural Information Processing Systems 35: Annual Conference on Neural Information Processing Systems 2022, NeurIPS 2022, New Orleans, LA, USA, November 28 - December 9, 2022*, 2022. URL http://papers.nips.cc/paper_files/paper/2022/hash/8644353f7d307baaf29bc1e56fe8e0ec-Abstract-Datasets_and_Benchmarks.html.

E. J. Gardiner, J. D. Holliday, O. Caroline, and W. Peter. Effectiveness of 2D fingerprints for scaffold hopping. *Future Medicinal Chemistry*, 3(4):405–414, 03 2011. ISSN 1756-8919.

W. Hamilton, Z. Ying, and J. Leskovec. Inductive representation learning on large graphs. In *Advances in Neural Information Processing Systems*, volume 30, page 1025–1035, 2017.

W. Hu, B. Liu, J. Gomes, and et al. Strategies for pre-training graph neural networks. In *International Conference on Learning Representations (ICLR)*, 2020a.

W. Hu, B. Liu, J. Gomes, M. Zitnik, P. Liang, V. Pande, and J. Leskovec. Strategies for pre-training graph neural networks. In *International Conference on Learning Representations*, 2020b.

J. J. Irwin, T. Sterling, M. M. Mysinger, E. S. Bolstad, and R. G. Coleman. Zinc: A free tool to discover chemistry for biology. *Journal of Chemical Information and Modeling*, 52(7):1757–1768, 2012. ISSN 1549-9596.

R. Irwin, S. Dimitriadis, J. He, and E. J. Bjerrum. Chemformer: a pre-trained transformer for computational chemistry. *Machine Learning: Science and Technology*, 3(1):015022, 2022.

A. Q. Jiang, A. Sablayrolles, A. Mensch, and et al. Mistral 7b, 2023.

S. Kim, J. Chen, T. Cheng, A. Gindulyte, J. He, S. He, Q. Li, B. A. Shoemaker, P. A. Thiessen, B. Yu, L. Zaslavsky, J. Zhang, and E. E. Bolton. PubChem 2023 update. *Nucleic Acids Research*, 51(D1):D1373–D1380, 2022. ISSN 0305-1048.

M. Kuhn, I. Letunic, L. J. Jensen, and P. Bork. The SIDER database of drugs and side effects. *Nucleic Acids Research*, 44 (D1):D1075–D1079, 10 2015. ISSN 0305-1048.

M. Lewis, Y. Liu, N. Goyal, and et al. BART: Denoising sequence-to-sequence pre-training for natural language generation, translation, and comprehension. In *Proceedings of the 58th Annual Meeting of the Association for Computational Linguistics*, pages 7871–7880, 2020.

G. Liu, J. Xu, T. Luo, and M. Jiang. Inverse molecular design with multi-conditional diffusion guidance, 2024.

S. Liu, H. Wang, W. Liu, J. Lasenby, H. Guo, and J. Tang. Pre-training molecular graph representation with 3D geometry. In *ICLR 2022 Workshop on Geometrical and Topological Representation Learning*, 2022.

Z. Liu, W. Zhang, Y. Xia, L. Wu, S. Xie, T. Qin, M. Zhang, and T.-Y. Liu. MolXPT: Wrapping molecules with text for generative pre-training. In *Proceedings of the 61st Annual Meeting of the Association for Computational Linguistics (Volume 2: Short Papers)*, pages 1606–1616, 2023.

Y. Luo, K. Yang, M. Hong, X. Y. Liu, and Z. Nie. MolFM: A Multimodal Molecular Foundation Model. *arXiv e-prints*, 2023.

Y. Luo, J. Zhang, S. Fan, and et al. BioMedGPT: An open multimodal large language model for biomedicine. *IEEE Journal of Biomedical and Health Informatics*, pages 1–12, 2024.

R. Ma and T. Luo. Pi1m: A benchmark database for polymer informatics. *Journal of Chemical Information and Modeling*, 60(10):4684–4690, 2020. doi: 10.1021/acs.jcim.0c00726.

I. F. Martins, A. L. Teixeira, L. Pinheiro, and A. O. Falcao. A bayesian approach to in silico blood-brain barrier penetration modeling. *Journal of Chemical Information and Modeling*, 52 (6):1686–1697, 2012. ISSN 1549-9596.

M. Mishra, M. Stallone, G. Zhang, and et al. Granite code models: A family of open foundation models for code intelligence, 2024.

D. L. Mobley and J. P. Guthrie. FreeSolv: a database of experimental and calculated hydration free energies, with input files. *Journal of Computer-Aided Molecular Design*, 28(7): 711–720, 2014. ISSN 1573-4951.

P. A. Novick, O. F. Ortiz, J. Poelman, A. Y. Abdulhay, and V. S. Pande. SWEETLEAD: an in silico database of approved drugs, regulated chemicals, and herbal isolates for computer-aided drug discovery. *PLOS ONE*, 8(11), 11 2013.

Open AI. GPT-4 technical report. https://arxiv.org/abs/2303.08774, 2023.

K. Papineni, S. Roukos, T. Ward, and W.-J. Zhu. BLEU: a method for automatic evaluation of machine translation. In *Proceedings of the 40th Annual Meeting on Association for Computational Linguistics*, ACL '02, page 311–318, 2002.

S. Preissner, K. Kroll, M. Dunkel, C. Senger, G. Goldsobel, D. Kuzman, S. Guenther, R. Winnenburg, M. Schroeder, and R. Preissner. SuperCYP: a comprehensive database on cytochrome p450 enzymes including a tool for analysis of cyp-drug interactions. *Nucleic Acids Research*, 38(Database issue): D237–D243, 2010.

C. Raffel, N. M. Shazeer, A. Roberts, and et al. Exploring the limits of transfer learning with a unified Text-to-Text Transformer. *The Journal of Machine Learning Research*, 21: 140:1–140:67, 2019.

R. Ramakrishnan, P. O. Dral, M. Rupp, and O. A. von Lilienfeld. Quantum chemistry structures and properties of 134 kilo molecules. *Scientific Data*, 1(1):140022, 2014. ISSN 2052-4463.

A. M. Richard, R. S. Judson, K. A. Houck, C. M. Grulke, P. Volarath, I. Thillainadarajah, C. Yang, J. Rathman, M. T. Martin, J. F. Wambaugh, T. B. Knudsen, J. Kancherla, K. Mansouri, G. Patlewicz, A. J. Williams, S. B. Little, K. M. Crofton, and R. S. Thomas. ToxCast chemical landscape: Paving the road to 21st century toxicology. *Chemical Research in Toxicology*, 29(8):1225–1251, 2016. ISSN 0893-228X.

M. Schlichtkrull, T. N. Kipf, P. Bloem, and et al. Modeling relational data with graph convolutional networks. In *The Semantic Web: 15th International Conference, ESWC*, page 593–607, 2018.

P. Schwaller, T. Laino, T. Gaudin, P. Bolgar, C. A. Hunter, C. Bekas, and A. A. Lee. Molecular transformer: A model for uncertainty-calibrated chemical reaction prediction. *ACS Central Science*, 5(9):1572–1583, 2019. ISSN 2374-7943.

G. Subramanian, B. Ramsundar, V. Pande, and R. A. Denny. Computational modeling of β-secretase 1 (bace-1) inhibitors using ligand based approaches. *Journal of Chemical Information and Modeling*, 56(10):1936–1949, 2016. ISSN 1549-9596.

F.-Y. Sun, J. Hoffmann, V. Verma, and J. Tang. Infograph: Unsupervised and semi-supervised graph-level representation learning via mutual information maximization. In *International Conference on Learning Representations (ICLR)*, 2020.

R. Taylor, M. Kardas, G. Cucurull, T. Scialom, A. S. Hartshorn, and et al. Galactica: A large language model for science. *ArXiv*, abs/2211.09085, 2022.

A. Thornton, L. Robeson, B. Freeman, and D. Uhlmann. Membrane database: Polymer gas separation. `https://research.csiro.au/virtualscreening/membrane-database-polymer-gas-separation-membranes/`, 2012.

H. Touvron, T. Lavril, G. Izacard, and et al. Llama: Open and efficient foundation language models. *CoRR*, abs/2302.13971, 2023a.

H. Touvron, T. Lavril, G. Izacard, X. Martinet, M.-A. Lachaux, T. Lacroix, B. Rozière, N. Goyal, E. Hambro, F. Azhar, A. Rodriguez, A. Joulin, E. Grave, and G. Lample. LLaMA: Open and efficient foundation language models, 2023b.

P. Veličković, G. Cucurull, A. Casanova, A. Romero, P. Liò, and Y. Bengio. Graph attention networks. *arXiv*, 2017. URL `https://arxiv.org/abs/1710.10903`.

P. Veličković, W. Fedus, W. L. Hamilton, P. Liò, Y. Bengio, and R. D. Hjelm. Deep graph infomax. In *International Conference on Learning Representations*, 2019.

Y. Wan, C.-Y. Hsieh, B. Liao, and S. Zhang. Retroformer: Pushing the limits of end-to-end retrosynthesis transformer. In *Proceedings of the 39th International Conference on Machine Learning*, volume 162 of *Proceedings of Machine Learning Research*, pages 22475–22490, 2022.

H. Wang, W. Li, X. Jin, K. Cho, H. Ji, J. Han, and M. D. Burke. Chemical-reaction-aware molecule representation learning. In *Proceedings of the International Conference on Learning Representations (ICLR)*, 2022a.

Y. Wang, J. Xiao, T. O. Suzek, J. Zhang, J. Wang, Z. Zhou, L. Han, K. Karapetyan, S. Dracheva, B. A. Shoemaker, E. Bolton, A. Gindulyte, and S. H. Bryant. PubChem's bioassay database. *Nucleic Acids Research*, 40(D1):D400–D412, 2011. ISSN 0305-1048.

Y. Wang, J. Wang, Z. Cao, and A. Barati Farimani. Molecular contrastive learning of representations via graph neural networks. *Nature Machine Intelligence*, 4(3):279–287, 2022b. ISSN 2522-5839.

D. Weininger. SMILES, a chemical language and information system. 1. introduction to methodology and encoding rules. *Journal of Chemical Information and Computer Sciences*, 28 (1):31–36, 1988.

D. S. Wishart, Y. D. Feunang, A. C. Guo, E. J. Lo, A. Marcu, J. R. Grant, T. Sajed, D. Johnson, C. Li, Z. Sayeeda, N. Assempour, I. Iynkkaran, Y. Liu, A. Maciejewski, N. Gale, A. Wilson, L. Chin, R. Cummings, D. Le, A. Pon, C. Knox, and M. Wilson. DrugBank 5.0: a major update to the drugbank database for 2018. *Nucleic Acids Research*, 46(D1):D1074–D1082, 11 2017. ISSN 0305-1048.

Z. Wu, B. Ramsundar, E. N. Feinberg, J. Gomes, C. Geniesse, A. S. Pappu, K. Leswing, and V. Pande. MoleculeNet: a benchmark for molecular machine learning. *Chemical Science*, 9:513–530, 2018.

C. Xu, D. Guo, N. Duan, and J. McAuley. Baize: An open-source chat model with parameter-efficient tuning on self-chat data. In *Proceedings of the 2023 Conference on Empirical Methods in Natural Language Processing*, pages 6268–6278, 2023.

Z. Xu, J. Li, Z. Yang, S. Li, and H. Li. SwinOCSR: end-to-end optical chemical structure recognition using a swin transformer. *Journal of Cheminformatics*, 14(1):41, 2022. ISSN 1758-2946.

Y. You, T. Chen, Y. Sui, T. Chen, Z. Wang, and Y. Shen. Graph contrastive learning with augmentations. In *Advances in Neural Information Processing Systems*, volume 33, pages 5812–5823, 2020.

B. Zdrazil, E. Felix, F. Hunter, E. Manners, J. Blackshaw, S. Corbett, M. de Veij, H. Ioannidis, D. Lopez, J. Mosquera, M. Magarinos, N. Bosc, R. Arcila, T. Kizilören, A. Gaulton, A. Bento, M. Adasme, P. Monecke, G. Landrum, and A. Leach. The ChEMBL database in 2023: a drug discovery platform spanning multiple bioactivity data types and time periods. *Nucleic Acids Research*, 52(D1):D1180–D1192, 2024.

A. Zeng, X. Liu, Z. Du, Z. Wang, H. Lai, M. Ding, Z. Yang, Y. Xu, W. Zheng, X. Xia, W. L. Tam, Z. Ma, Y. Xue, J. Zhai, W. Chen, P. Zhang, Y. Dong, and J. Tang. GLM-130B: An open bilingual pre-trained model, 2023.

Z. Zeng, Y. Yao, Z. Liu, and M. Sun. A deep-learning system bridging molecule structure and biomedical text with comprehension comparable to human professionals. *Nature Communications*, 13(1):862, 2022. ISSN 2041-1723.

G. Zhou, Z. Gao, Q. Ding, H. Zheng, H. Xu, Z. Wei, L. Zhang, and G. Ke. Uni-Mol: A universal 3D molecular representation learning framework. In *International Conference on Learning Representations*, 2023.

J. Zhu, Y. Xia, L. Wu, S. Xie, W. Zhou, T. Qin, H. Li, and T.-Y. Liu. Dual-view molecular pre-training. In *Proceedings of the 29th ACM SIGKDD Conference on Knowledge Discovery and Data Mining*, KDD '23, page 3615–3627, 2023. ISBN 9798400701030.
